# Familial risks of hospital treated osteoporosis and fractures in first-, second- and third-degree relatives - A Swedish nationwide real-world family study

**DOI:** 10.1371/journal.pone.0358074

**Published:** 2026-09-11

**Authors:** Christian Anker-Hansen, MirNabi Pirouzifard, Jan Sundquist, Kristina Sundquist, Bengt Zöller

**Affiliations:** 1 Center for Primary Health Care Research, Lund University/Region Skåne, Malmö, Sweden; 2 University Clinic Primary Care, Skåne University Hospital, Region Skåne, Sweden; Showa University, JAPAN

## Abstract

**Objective:**

Osteoporosis (OP) is a common degenerative disorder. This is the first real-world nationwide study aimed to determine the familial risks of hospital treated OP and fractures in first-, second- and third-degree relatives.

**Design:**

The Swedish Multigeneration register was linked to the National Patient Register to investigate the familial risks of OP between 1997 and 2018. Offspring born to Swedish parents were included. The adjusted familial hazard ratios (afHRs) with 95% confidence interval (CI) were determined for hospital treated OP and fractures at common osteoporotic fracture sites among twins, full-siblings, half-siblings, and cousins. There was no information about zygosity of the twins. Adjustments were made for birth year, sex, educational level, and comorbidities.

**Results:**

6,548,562 individuals (48.77% women) were included with a mean age of 41 years (range 0−87 years) at the end of follow-up. 57,869 (0.88%) individuals were affected with hospital treated OP, and 403,660 (6.16%) individuals with fractures. The afHRs were increased for both OP and fractures in twins, full-siblings, half-siblings, and cousins. The afHR for OP were for twins 2.92 (95%CI 2.32–3.67), full-siblings 2.22 (95%CI 2.14–2.31), half-siblings 1.74 (95%CI 1.55–1.94), cousins 1.81 (95%CI 1.55–2.11). Age-stratified analysis showed age-dependent familial associations with highest afHR in young individuals (<20 years): among full-siblings afHR was 125.70 (95%CI 59.44–265.84) for OP and 2.34 (95%CI 2.28–2.39) for fractures.

**Conclusions:**

Heredity is associated with risk of hospital treated OP and fractures in the Swedish population. The familial risk of hospital treated OP or fractures is related to the genetic closeness of the affected relative(s).

## Introduction

Osteoporosis (OP) is a degenerative bone disease characterised by impaired bone remodelling, resulting in a gradual loss of skeletal bone and low bone mineral density (BMD) [1]. It has been estimated that one-third of women and one out of eight men over the age of 50 years have OP [2]. OP incidence increases with age, and the gradual loss of sex hormones with age, especially loss of oestrogen in women after menopause, puts postmenopausal women at greater risk [3]. Other risk factors include nutritional deficiencies, weight loss, sedentary lifestyle, smoking, high alcohol consumption, white ethnic background, and medications such as oral corticosteroids [4]. OP has also been shown to exhibit polygenetic inheritance, i.e., OP is a complex disorder [5].

OP is a silent disease, and first symptom is often fragility fractures, i.e., fractures due to low-energy trauma [6]. Hip-, pelvic-, vertebral-, proximal humeral-, and distal radius fractures are considered the main OP related fractures [7].

OP and OP related fractures are increasing globally due to longer life expectancy and more sedentary lifestyles, especially during young age when we form our skeleton and build up our peak bone mass (PBM) [8]. OP related fractures may lead to substantial medical costs and suffering for those affected [9]. OP can be prevented to some degree with calcium- and vitamin D rich diets as well as regular exercise [10,11]. There are now effective treatments for OP, including antiresorptive drugs that inhibit osteoclast mediated bone resorption and anabolic agents that stimulate osteoblast mediated bone formation [12]. However, these treatments are often commenced after the patient had their first OP related fracture [13]. Moreover, only a minority of OP patients receive any treatment at all [13]. In this aspect, it is an advantage to be able to predict an individual’s OP risk before having the first OP related fracture. The most widely used tool for OP prediction is the “Fracture Risk Assessment Tool”, abbreviated as the FRAX questionnaire [14]. However, FRAX with BMD requires DXA access, which may limit use in some settings.

Familial susceptibility to OP results from both genetic and environmental factors and their interaction [5,15]. It has been shown that between 50–85% of the variance in peak bone mass is genetically determined, depending on skeletal site and age [16]. Many aspects of the OP risk are genetically determined, such as femoral neck geometry [17], muscle strength [18], bone turnover markers [19], and body mass index [20]. In women, the age of menopause has been shown to be genetically determined, thus explaining some of the risk of OP [21]. In one study concerning hereditary patterns of patients with OP, 45% of cases reported a family history of OP [22]. However, the familial cases did not have an earlier age of onset and disease severity was like that of non-familial cases [22]. In another study of osteoporotic middle-aged men, low bone density in the lumbar spine was found in 54.8% of the relatives of osteoporotic patients versus 17.4% of the control subjects without OP with a familial risk of 3.2 [23]. In one study of male and female twins there was evidence of a genetic contribution to bone loss [24],, but in a male twin study, no such evidence was observed [25]. These conflicting results regarding the importance of heredity of OP call for more and larger studies of the familial aggregation of OP.

Another aspect of heredity to OP is whether fractures at common osteoporotic fracture sites aggregates in families, since fractures are often the first clinical manifestation of OP. Family history of fracture has been shown to be a risk factor for fractures independent of bone mineral density [26]. The explanation for this could be that the predisposition could be influenced by bone turnover and skeletal disposition, as well as risk factors for falling, such as cognitive function, neuromuscular control, and eyesight [16]. The genetic contribution to OP and fractures diminishes with age as environmental factors become more important [16]. This is a typical pattern for complex traits [27]. In a Swedish twin study, the heritability of hip fractures was 68% in twins under the age of 65, 47% between 69 and 79 years, but only 3% after the age of 79 years [28]. However, rare early-onset OP may occur [29]. Loss-of-function variants in *LRP5* and *WNT1* may lead to early-onset OP [29]. Other early-onset OP has been linked to disturbed sphingolipid metabolism due to SGMS2 mutations or X-chromosomal OP caused by PLS3 gene mutations affecting especially males [29]. Early‑onset OP has also been described to be a polygenic disorder due to an increased burden of common fracture risk alleles compared to the general population [29]. Polygenic risk scores (PRS) for OP have previously been developed [30–33]. Using data from the UK Biobank, a large genome-wide association study (GWAS) identified 1,362 independent single-nucleotide polymorphisms (SNPs) mapping to 899 loci, of which 613 were novel. Based on these findings, a PRS was constructed that demonstrated substantial heritability for BMD and OP, whereas its predictive capacity was comparatively lower for fracture outcomes [30].

In the era of GWAS and whole genome sequencing (WGS) the use of family studies may be questioned [34,35]. However, most diseases are the result of the interactions of multiple genes and environmental factors [34,35]. Family history captures these interactions and is free to use for the patient [34,35]. Moreover, while genetic tests and precision medicine have advanced rapidly, family history remains the “first genetic test.” It provides actionable clues that help prioritize individuals for genomic screening [34,35].

We aimed to determine the familial risks of hospital treated OP and fractures at common osteoporotic sites in a large nationwide population-based cohort in Sweden using the world’s largest familial database: the Swedish multigeneration register with linkage to the Swedish National Patient register (NPR) [36–39]. This is, to the best of our knowledge, the first real-world nationwide family study with determination of familial risk for hospital treated OP and fractures among twins, full-siblings, half-siblings, and cousins, i.e., first-, second-, and third-degree relatives. All age groups were included to cover also early-onset OP [29].

## Method

### Study population

We used several Swedish nationwide registers to study the familial risks of hospital treated OP [36–39]. Statistics Sweden, and the National Board of Health and Welfare maintain the registers used in the study. The Swedish personal identity number (PIN) is issued to all residents in Sweden and was used by Statistics Sweden to connect individual-level data from different registers [36–39]. To preserve integrity, the PIN were replaced by Statistics Sweden with pseudonymised serial numbers. The Swedish Population Register was linked to the Multigeneration Register to identify twins (but with no information about zygosity), full-siblings, half-siblings, and cousins. The Swedish Multigenerational Register provides reliable data on index cases born on January 1, 1932 and onwards. The follow-up time was until December 31, 2018. Linkage was then made to the NPR, including all hospital discharge diagnoses in Sweden from 1987 onwards. International Classification of Disease 10^th^ edition (ICD-10) started on January 1, 1997, and from this date, data on OP diagnoses and fractures at common osteoporotic fracture sites diagnoses were collected. Only ICD-10 diagnoses were used. The NPR also includes hospital outpatient diagnoses from 2001 to 2018; however, the registers do not include diagnoses from primary health care. Thus, the study includes data on hospital treated OP and fractures in patients born between 1932–2018, and being diagnosed 1997 and onwards. All age groups were included to cover also early-onset OP [29].

Inclusion criteria were individuals born in Sweden to Swedish born parents according to the Swedish population register. The reason for this was to provide secure details on kinship and have both biological parents be obligatorily known. Exclusion criteria were individuals not born in Sweden or with one or both parents born outside of Sweden. Individuals born before Jan 1, 1932, were also excluded as kinship is not defined in the Multigeneration Register for these individuals [36],].

We defined four groups of relative pairs: twins (siblings born on the same date), full-siblings, half-siblings, and cousins. We have no information about the zygosity of the included twins.

The same person was allowed to be included in more than one family relationship and in more than one type of fracture. We used double-entry to solve the problem as to which relative’s trait should be used as the dependent, and which as the independent variable [40,41]. In the analyses, each relative was thus entered twice – once as the first relative (dependent variable) and once as the second relative in a pair (explanatory variable or predictor for relative history of OP or fracture). This means that each relative is entered twice in the data, and each member of a relative pair provides once the dependent and once the explanatory variable as previously described. While the consistency of the regression estimates for heritability and environmental influences is not affected by double entry, the standard errors (SEs) of the coefficients are biased and need to be adjusted [40,41]. All possible relative pairs in a family were considered, and we used robust standard error to take into account non-independence between cases [40,41]. In the Swedish Population Register, we identified all relative pairs, i.e.,149,002 double-entry twins, 9,991,510 double-entry full-siblings, 2,839,532 double-entry half-siblings and 25,851,018 double-entry cousins (Tables 2 and 3). All possible relative pairs were ascertained in a family.

**Table 2:** Hazard ratios (HRs) with 95% confidence intervals (95% CIs) for patients with osteoporosis (OP) compared with their non-affected relatives.

Variable	Person-years, No.	Cases, No./Persons at risk, No.	Incidence rate, cases/1000 person-years	Incidence rate ratio (95%CI)	HR(95% CI)		
					Model 1	Model 2	Model 3
**Twins** (n = 149002)							
Non-osteoporosis	2646387	1290/ 147534	0.49 (0.46-0.51)	1 [Reference]	1 [Reference]	1 [Reference]	1 [Reference]
Osteoporosis	28196	178/1468	6.31 (5.45-7.31)	12.95*** (11.07-15.15)	11.73*** (9.38-14.66)	3.15*** (2.50-3.96)	2.92*** (2.32-3.67)
**Siblings** (n = 9991510)							
Non-osteoporosis	191475456	105936/9878006	0.55 (0.55-0.56)	1 [Reference]	1 [Reference]	1 [Reference]	1 [Reference]
Osteoporosis	2256361	7568/113504	3.35 (3.28-3.43)	6.06*** (5.92-6.21)	5.67*** (5.46-5.89)	2.32*** (2.24-2.541)	2.22*** (2.14-2.31)
**Half-siblings** (n = 2839532)							
Non- osteoporosis	54441042	13688/2825436	0.25 (0.25-26)	1 [Reference]	1 [Reference]	1 [Reference]	1 [Reference]
Osteoporosis	291461	408/14096	1.40 (1.27-1.54)	5.57*** (5.05-6.14)	5.21*** (4.68-5.79)	1.76*** (1.58-1.96)	1.74*** (1.55-1.94)
**Cousins** (n = 25851018)							
Non-osteoporosis	494426955	38097/25812735	0.08 (0.08-0.08)	1 [Reference]	1 [Reference]	1 [Reference]	1 [Reference]
Osteoporosis	812539	186/38283	0.23 (0.20-0.26)	2.97*** (2.57-3.43)	2.82*** (2.42-3.29)	1.90*** (1.63-2.21)	1.81*** (1.55-2.11)

HR, Hazard ratio. Model 1 crude. Model 2 adjusted for sex, education and birth year. Model 3 adjusted additionally for obesity, chronic obstructive pulmonary disease (COPD), alcohol use disorder, rheumatoid arthritis (RA), polymyalgia rheumatica (PMR) giant cell arthritis (GCA), inflammatory bowel disease (IBD), and asthma. All calculations were based on double entry. Significance levels: * p<0.05, ** p<0.01, *** p<0.001

**Table 3:** Hazard ratios (HRs) with 95% confidence intervals (95% CIs) for patients with fractures at common osteoporotic fracture sites (=fractures at OP sites) compared with their non-affected relatives.

Variable	Person-years, No.	Cases, No./Persons at risk, No.	Incidence rate, cases/1000 person-years	Incidence rate ratio (95%CI)	HR(95% CI)		
					Model 1	Model 2	Model 3
**Twins** (n = 149002)							
Non-osteoporosis fracture	2417888	8032 /139718	3.32 (3.25-3.40)	1 [Reference]	1 [Reference]	1 [Reference]	1 [Reference]
Osteoporosis fracture	177370	1252/ 9284	7.06 (6.68-7.46)	2.13*** (2.00-2.26)	2.07*** (1.90-2.25)	1.81*** (1.66-1.97)	1.76*** (1.62-1.92)
**Siblings** (n = 9991510)							
Non-osteoporosis fracture	174688597	609116 /9303086	3.49 (3.48-3.50)	1 [Reference]	1 [Reference]	1 [Reference]	1 [Reference]
Osteoporosis fracture	13381225	79308 /688424	5.93 (5.89-5.97)	1.70*** (1.69-1.71)	1.66*** (1.65-1.68)	1.47*** (1.45-1.48)	1.44*** (1.43-1.46)
**Half-siblings** (n = 2839532)							
Non-osteoporosis fracture	49868762	157604/2669634	3.16 (3.14-3.18)	1 [Reference]	1 [Reference]	1 [Reference]	1 [Reference]
Osteoporosis fracture	3250327	12294/169898	3.78 (3.72-3.85)	1.20*** (1.18-1.22)	1.19*** (1.17-1.21)	1.15*** (1.13-1.17)	1.14*** (1.12-1.16)
**Cousins** (n = 25851018)							
Non-osteoporosis fracture	458833521	1140173/24643182	2.48 (2.48-2.49)	1 [Reference]	1 [Reference]	1 [Reference]	1 [Reference]
Osteoporosis fracture	23585247	67663/1207836	2.87 (2.85-2.89)	1.16*** (1.15-1.16)	1.15*** (1.14-1.16)	1.15*** (1.14-1.16)	1.15*** (1.14-1.16)

HR, Hazard ratio. Model 1 crude. Model 2 adjusted for sex, education and birth year. Model 3 adjusted additionally for obesity, chronic obstructive pulmonary disease (COPD), alcohol use disorder, rheumatoid arthritis (RA), polymyalgia rheumatica (PMR), giant cell arthritis (GCA), inflammatory bowel disease (IBD), and asthma.. All calculations were based on double entry. Significance levels: * p<0.05, ** p<0.01, *** p<0.001.

### Definition of hospital treated OP and fractures at common osteoporotic fracture sites

ICD-10 started on January 1, 1997, and from this date, data on hospital treated OP diagnose and fractures at common osteoporotic fracture sites diagnoses were collected. All patients with main or secondary ICD-10 codes in NPR for OP diagnoses were used, i.e., osteoporosis with fracture (M80) and osteoporosis without fracture (M81). We also included hospital treated fractures at common osteoporotic fracture sites (i.e., possible OP related fractures), in a separate analysis. The included fractures at common osteoporotic fracture sites were hip fractures (S72), lumbar and thoracic vertebral fractures and ribs (S32, S22), pubis pelvic fractures (S325), proximal humeral fractures (S422), and distal radius fractures (S525). All patients with main or secondary ICD-10 fracture codes in NPR were included.

### Main outcomes

Main outcomes are hospital treated OP diagnosis (ICD-10 codes M80, M81) or fractures at common osteoporotic fracture sites (ICD-10 codes S72, S32, S22, S325, S422, S525). An extended analysis was also performed among full-siblings using eight different fracture types (S22, S32, S72, S422, S423, S525, S825, and S826). S423 denotes fracture of the shaft of humerus, S825 is fracture of medial malleolus, and S826 is fracture of lateral malleolus, In a further analysis among full-siblings with these 8 fracture types only those due to falls were included (ICD-10 codes W00-W19).

### Validation of ICD-10 codes M80 and M81

Diagnoses of OP based on hospital records (ICD-10 codes M80 and M81) have not been specifically validated. However, the Swedish NPR is generally considered to have high validity [38,39]. To indirectly support the diagnostic accuracy, prescription data were examined among affected full siblings using the Swedish Prescribed Drug Register in a subanalysis [42].

### Main predictors

Main predictors are hospital treated OP diagnosis (ICD-10 codes M80, M81) or fractures at common osteoporotic fracture sites (ICD-10 codes S72, S32, S22, S325, S422, S525) in twins, full-siblings, half-siblings, or cousins. The exposure OP diagnosis or fractures is defined over the entire follow-up period.

### Adjusting variables

Birth year was defined as the year of birth in the Swedish Population Register. Sex was defined as male or female. Education level was defined as level 1 = primary school only (9 years in Sweden) or missing value, level 2 = 10–11 years, or level 3 = more than 11 years of education.

Comorbidities used for adjustment were defined by ICD-10 codes in the NPR; obesity (E66), chronic obstructive pulmonary disease (COPD) (J41-J44), alcohol use disorder (F10), rheumatoid arthritis (RA) (M05, M06), polymyalgia rheumatica (PMR) (M353) including giant cell arthritis (GCA) with or without PMR (M315 or M316), inflammatory bowel disease (IBD) (K50, K51), and asthma (J45) [4].

### Statistical analysis

Baseline for study participants was defined as the date of inclusion, i.e., 1 January 1997, or date of birth. Follow-up time was the time from baseline to event of OP or fractures at common osteoporotic fracture sites, emigration, death, or end of follow-up, whichever came first.

The average genetic resemblance for twins, both monozygotic and dizygotic, was determined as 0.66 with Weinberg’s differential method. Each relative pair was assigned their average genetic resemblance (i.e., 0.66 for twin pairs, 0.5 for full-sibling pairs, 0.25 for half-sibling pairs, and 0.125 for cousin pairs).

Incidence rates for OP and fractures at common osteoporotic fracture sites were defined as the number of events divided by the person‐time at risk. The familial incidence rate ratio (IRR) between two incidence densities is calculated as the rate in the exposed population divided by the rate in those unexposed. OP‐free survival curves were constructed according to the Kaplan–Meier method to compare individuals with and without a relative history of respective OP. For comparison of two curves, the log‐rank test, resulting in a test statistic with a χ^2^ distribution and 1 *df*, was used. The adjusted familial Hazard ratios (afHRs) with a confidence interval (CI) of 95% between twins, full‐siblings, half-siblings, and cousins was also used. For comparison, parent-offspring risks were also calculated but without double entry. OP events were estimated using competing-event analysis according to Fine and Gray [43]. Death and emigration were competing events. Model 1 was crude. Model 2 was adjusted for year of birth, sex, and level of education. Model 3 was additionally adjusted for obesity, COPD, alcohol use disorder, RA, PMR and GCA, IBD and asthma. In an additional analysis the covariates were added stepwise: model A was crude, model B was adjusted for birthyear, model C was adjusted for sex, model D was adjusted for birthyear, sex, and education, and model E was additionally adjusted for obesity, COPD, alcohol use disorder, RA, PMR and GCA, IBD, and asthma. Familial HRs for OP and fractures at common osteoporotic fracture sites were calculated for relatives of individuals who had a diagnosis of OP compared with relatives of individuals unaffected by OP as the reference group.

Among full-siblings an age stratified analysis (<20, 20–39, 40–49, 50–59, 60–69, 70–79, and 80 years and above) was performed. Moreover, among full-siblings an analysis was performed using 8 different fracture types (S22, S32, S72, S422, S423, S525, S825, and S826) instead of five fracture types (S22, S32, S422, S525, S72). In a further analysis among full-siblings patients with fracture due to falls were included (W00-W19).

Statistical significance was set at *P* < 0.05, and all tests were two-tailed. Data were analysed using SAS version 9.4 (SAS Institute, USA). All data was extracted on 02/12/2024.

### Ethical approval

The regional ethical committee at Lund University approved the study (approval nos. 2012/795, approved 2013-02-06), and all the protocols were conducted in accordance with the Helsinki Declaration and the Data Registry Inspection in Stockholm.

## Results

### Descriptives

A total of 6,548,562 individuals met the inclusion criteria (Table 1). In the study population, 48.77% were females and 51.23% were males with a mean age of 41.16 years (range of 0–87 years) at the end of follow-up (Table 1). 45.87% had an education level of more than 11 years.

**Table 1:** Characteristics of patients with osteoporosis (OP) and fractures at common osteoporotic fracture sites (=fractures at OP sites) in the whole study sample (unique individuals).

	All individuals n = 6548562 (100%)	No OP n = 6490693 (99.12)	OP n = 57869 (0.88)	*P-value*	No Fractures at OP sites n = 6144902 (93.84)	Fractures at OP sites n = 403660 (6.16)	*P-value*
**Sex** ^£^							
Male	3354992 (51.23)	3344927 (51.53)	10065 (17.39)	<0.001	3154155 (51.33)	200837 (49.75)	<0.001
Female	3193570 (48.77)	3145766 (48.47)	47804 (82.61)		2990747 (48.67)	202823 (50.25)	
**Education** ^£^							
> 11y	3003763 (45.87)	2987004 (46.02)	16842 (29.10)	<0.001	2861198 (46.56)	155281 (38.47)	<0.001
Year of birth*							
Mean (SD)	1976.53 (23.22)	1976.80 (23.13)	1946.83 (11.53)	<0.001	1977.25 (23.09)	1965.58 (22.47)	<0.001
Median (IQR)	1976 (1957-1995)	1976 (1958-1996)	1944 (1939-1951)		1977 (1958-1996)	1961 (1945-1989)	
(Range)	(1932-2018)	(1932-2018)	(1932-2016)		(1932-2018)	(1932-2018)	
Age at end follow-up*							
Mean (SD)	41.16 (22.71)	41.22(22.72)	64.38 (12.36)	<0.001	40.77(22.68)	42.78 (23.73)	<0.001
Median (IQR)	42.08 (22.24-60.25)	41.91 (22.75-60.16)	66.39 (58.88-72.76)		41.50 (22.16-59.50)	48.08 (16.74-63.15)	
(Range)	(0.00-87.00)	(0.00-87.00)	(0.20-86.71)		(0.00-87.00)	(0.02-86.90)	
**Comorbidities** ^£^ **; %**							
Obesity	2.96	2.95	3.84	<0.001	2.91	3.76	<0.001
COPD	1.67	1.54	16.03	<0.001	1.42	5.50	<0.001
Alcohol use disorder	3.15	3.13	5.43	<0.001	2.82	8.14	<0.001
RA	0.86	0.78	9.73	<0.001	0.79	2.02	<0.001
PMR and GCA	0.43	0.38	5.84	<0.001	0.38	1.16	<0.001
IBD	1.15	1.13	4.17	<0.001	1.12	1.62	<0.001
Asthma	6.47	6.44	10.23	<0.001	6.39	7.72	<0.001

Age at end of follow-up: death, migration, osteoporosis, osteoporosis fracture diagnosis or end of study period on December 31, 2018, whichever came first; SD, standard deviation; IQR, interquartile range.

*P-value: Mann-Whitney test.

£*P-value*: Chi-square test.

Abbreviations: chronic obstructive pulmonary disease (COPD), rheumatoid arthritis (RA), polymyalgia rheumatica (PMR), giant cell arthritis (GCA), and inflammatory bowel disease (IBD).

Out of the total study population, 57,869 individuals (0.88%) had hospital treated OP and 403,660 individuals had fractures at common osteoporotic fracture sites (6.16%). Out of the individuals with OP, 82.61% were females, the mean age was 64.38 years, and 29.10% had an education level of more than 11 years. Out of patients with fractures at common osteoporotic fracture sites, 50.25% were females, the mean age was 42.78 years, and 38.47% had an education level of more than 11 years. All comorbidities were more common in patients with OP or fractures at common osteoporotic fracture sites compared with non-affected individuals (Table 1).

### Familial risk of hospital treated OP

#### Twins.

**Fig 1:**
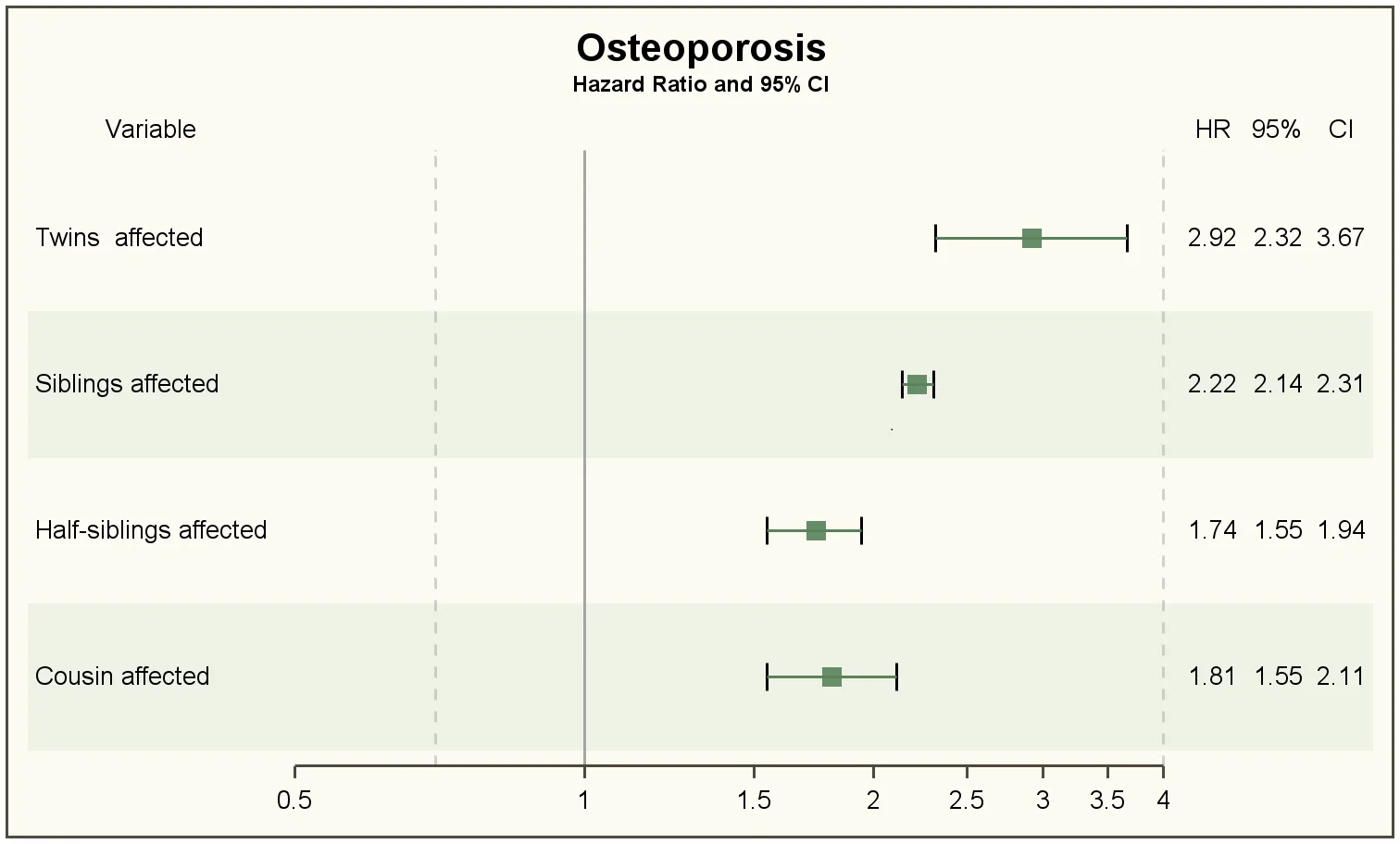
Forest plots of adjusted familial hazard ratios (HR) for osteoporosis (OP) among twins, full siblings, half-siblings, and cousins. Abbreviations: CI = confidence interval.

Out of the 146,642 unique twins (149,002 double-entry twins), 49.95% were female, and the mean age was 37.52 years (S1 Table and Table 2). Among the twins, 1461 subjects had OP (1.00%) and the mean age in this group was 65.22 years, with 83.27% being female (S1 Table). The fully afHR for twins was 2.92 (95%CI 2.32–3.67) (Table 2 and Fig 1).

#### Full-siblings.

Out of the 5,714,784 unique full-siblings (9,991,510 double-entry full-siblings), 48.73% were female, and the mean age was 42.43 years (S2 Table and Table 2). Among the full-siblings 54,306 subjects had OP (0.95%), and the mean age in this group was 64.82 years, with 82.82% being female (S2 Table). The fully afHR for full-siblings was 2.22 (95%CI 2.14–2.31) (Table 2 and Fig 1).

#### Half-Siblings.

Out of the 1,375,039 unique half-siblings (2,839,532 double-entry half-siblings), 48.94% were female, and the mean age was 36.48 years (S3 Table and Table 2). Among the half-siblings, 6687 subjects had OP (0.49%) and the mean age in this group was 58.05 years, with 79.92% being female (S3 Table). The fully afHR for half-siblings was 1.74 (95%CI 1.55–1.94) (Table 2 and Fig 1).

#### Cousins.

Out of the 4,149,511 unique cousins (25,851,018 double entry cousins), 48.62% were female, and the mean age was 29.95 years (S4 Table and Table 2). Among the cousins, 5806 subjects had OP (0.14%) and the mean age in this group was 40.79 years, with 70.07% being female (S4 Table). The fully afHR for cousins was 1.81 (95%CI 1.55–2.11) (Table 2 and Fig 1).

### Familial risk of fractures at common hospital treated OP fracture sites

#### Twins.

**Fig 2:**
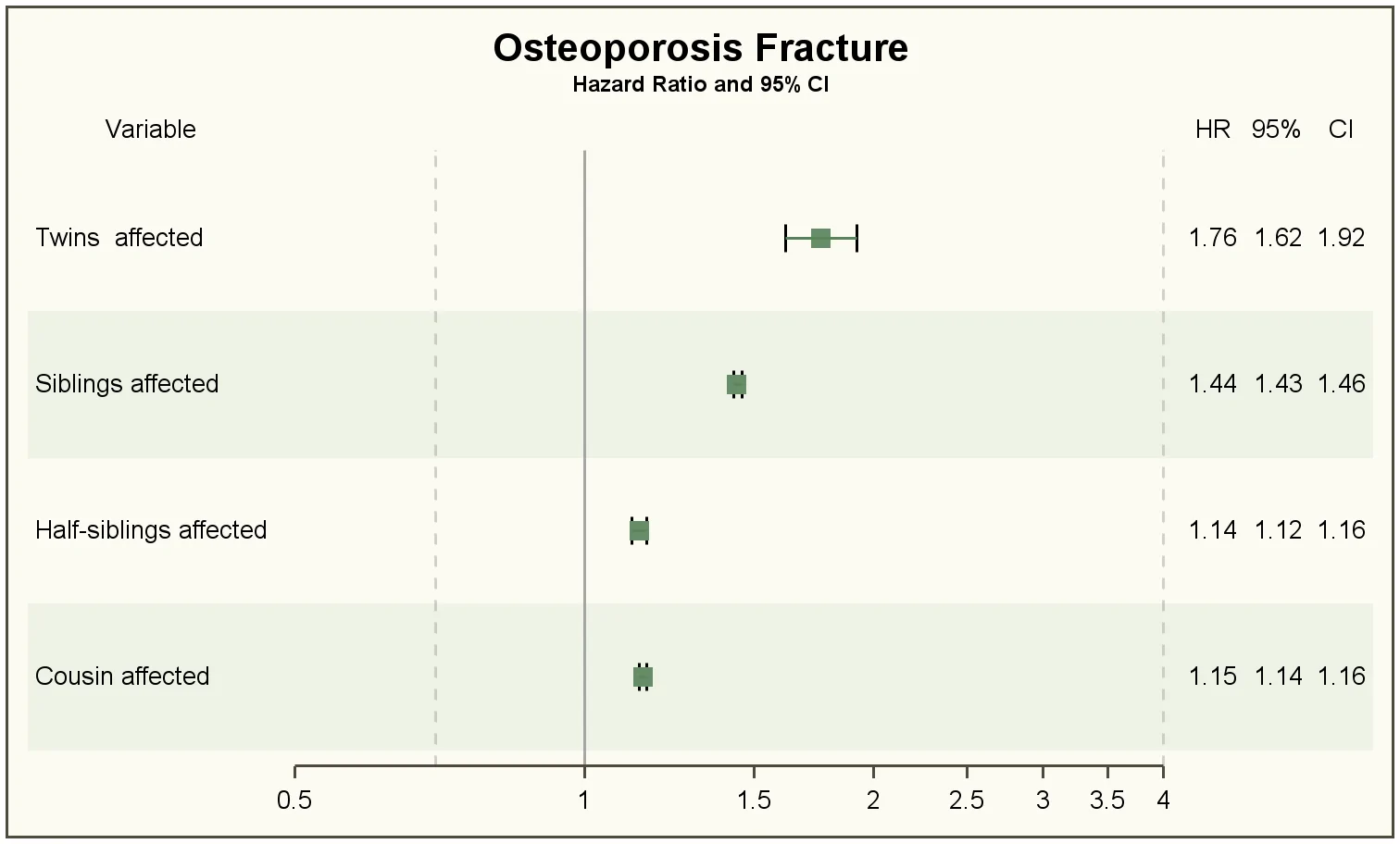
Forest plots of adjusted familial hazard ratios (HR) for fractures at common osteoporotic fracture sites (=fractures at OP sites) among twins, full‐siblings, half-siblings, and cousins. CI = confidence interval.

Out of the 146,642 unique twins (149,002 double-entry twins), 49.95% were female and the mean age was 37.52 years (S1 Table and Table 3). Among the twins, 9138 subjects had fractures at common osteoporotic fracture sites (6.24%), and the mean age in this group was 41.59 years, with 52.55% being female (S1 Table). The fully afHR for twins was 1.76 (95%CI 1.62–1.92) (Table 3 and Fig 2).

#### Full-siblings.

Out of the 5,714,784 unique full-siblings (9,991,510 double-entry full-siblings), 48.73% were female, and the mean age was 42.43 years. (S2 Table and Table 3) Among the full-siblings, 362,370 subjects had fractures at common osteoporotic fracture sites (6.34%) and the mean age in this group was 43.94 years, with 50.72% being female (S2 Table). The fully afHR for full-siblings was 1.44 (95%CI 1.43–1.46) (Table 3 and Fig 2).

#### Half-siblings.

Out of the 1,375,039 unique half-siblings (2,839,532 double-entry half-siblings), 48.94% were female, and the mean age was 36.48 years (S3 Table and Table 3). Among the half-siblings, 80,561 subjects had fractures at common osteoporotic fracture sites (5.86%), and the mean age in this group was 33.21 years, with 45.44% being female (S3 Table). The fully afHR for half-siblings was 1.14 (95%CI 1.12–1.16) (Table 3 and Fig 2).

#### Cousins.

Out of the 4,149,511 unique cousins (25,851,018 double entry cousins), 48.62% were female, and the mean age was 29.95 years (S4 Table and Table 3). Among the cousins, 185,778 subjects had fractures at common osteoporotic fracture sites (4.48%), and the mean age in this group was 22.54 years, with 41.55% being female (S4 Table). The fully afHR for cousins was 1.15 (95%CI 1.14–1.16) (Table 3 and Fig 2).

### Age-specific familial HRs of hospital treated OP and common osteoporitc fracture sites

Age-specific hazard ratios (HRs) for hospital-treated osteoporosis (OP) and major osteoporotic fracture sites among full siblings are presented in Tables 4 and 5. A pronounced age-dependent gradient in familial risk was observed, particularly for OP. The familial HRs for OP were markedly elevated at younger ages, decreasing progressively with increasing age, ranging from 125.70 (95% CI 59.44–265.84) among individuals younger than 20 years to 1.61 (95% CI 1.44–1.80) among those aged 80 years and older (Table 4).

**Table 4:** Age-specific familial hazard ratios (HRs) for the association between familial exposure among full-siblings and osteoporosis (OP) (ICD-10 codes M80 or M81).

Age group (years)	N individuals	Events (n)	HRs (95% CI)					
			Model 1		Model 2		Model 3	
<20	1651375	679	132.40	(64.20-273.05)	129.21	(61.74-270.40)	125.70	(59.44-265.84)
20–39	2301374	2997	25.47	(18.56-34.94)	25.38	(18.46-34.88)	24.68	(17.95-33.91)
40–49	1143506	4670	18.21	(15.09-21.97)	17.87	(14.82-21.55)	17.58	(14.57-21.21)
50–59	1472477	19054	8.23	(7.62-8.89)	7.95	(7.35-8.59)	7.31	(6.76-7.90)
60–69	1662876	42459	3.78	(3.60-3.97)	3.74	(3.56-3.92)	3.32	(3.15-3.49)
70–79	1434508	37769	2.27	(2.16-2.39)	2.28	(2.16-2.39)	2.11	(2.01-2.23)
≥80	325394	5876	1.66	(1.48-1.85)	1.68	(1.50-1.87)	1.61	(1.44-1.80)

HR, Hazard ratio. Model 1 crude. Model 2 adjusted for sex and education. Model 3 adjusted additionally for obesity, chronic obstructive pulmonary disease (COPD), alcohol use disorder, rheumatoid arthritis (RA), polymyalgia rheumatica (PMR), giant cell arthritis (GCA), inflammatory bowel disease (IBD), and asthma. All calculations were based on double entry. Estimates are derived from models including an interaction between familial exposure and age groups (reference age group: 20–39 years).

**Table 5:** Age-specific familial hazard ratios (HRs) for the association between familial exposure and outcome in patients with common osteoporotic (OP) fractures (=fractures at OP sites, i.e., ICD-10 codes S22, S32, S422, S525, S72) compared with unaffected relatives.

Age group (years)	N individuals	Events (n)	HRs (95% CI)					
			Model 1		Model 2		Model 3	
<20	1760979	148319	2.37	(2.31-2.43)	2.36	(2.30-2.42)	2.34	(2.28-2.39)
20–39	2232710	77956	1.07	(1.04-1.11)	1.07	(1.03-1.12)	1.06	(1.02-1.10)
40–49	1167616	67770	2.10	(2.03-2.18)	2.04	(1.97-2.11)	1.99	(1.93-2.06)
50–59	1514305	129441	2.08	(2.04-2.13)	2.02	(1.97-2.06)	1.94	(1.90-1.98)
60–69	1665886	157893	1.70	(1.67-1.73)	1.67	(1.64-1.70)	1.62	(1.59-1.64)
70–79	1358327	92530	1.40	(1.37-1.43)	1.39	(1.36-1.42)	1.36	(1.33-1.39)
≥80	291687	14515	1.21	(1.15-1.27)	1.21	(1.15-1.27)	1.20	(1.14-1.26)

HR, Hazard ratio. Model 1 crude. Model 2 adjusted for sex and education. Model 3 adjusted additionally for obesity, chronic obstructive pulmonary disease (COPD), alcohol use disorder, rheumatoid arthritis (RA), polymyalgia rheumatica (PMR), giant cell arthritis (GCA), inflammatory bowel disease (IBD), and asthma. All calculations were based on double entry. Estimates are derived from models including an interaction between familial exposure and age groups (reference age group: 20–39 years).

A similar age-dependent pattern was evident for osteoporotic fracture sites, with the notable exception of individuals aged 20–39 years. For these fracture outcomes, the familial HRs declined with increasing age, ranging from 2.34 (95% CI 2.28–2.39) in individuals younger than 20 years to 1.20 (95% CI 1.14–1.26) in those aged 80 years and older (Table 5).

### Kaplan-Meier analysis, Cumulative Incidence Function, and log-log curves

S1-S6 Figs display the Kaplan-Meier analysis, cumulative incidence function (CIF), and log-log survival curves for OP and fractures at common osteoporotic fracture sites for familial and non-familial cases. The familial risk of OP and fractures at common osteoporotic fracture sites are displayed using Kaplan-Meier analysis for twins, full-siblings, half-siblings, and cousins, suggesting a relationship between OP-free survival and degree of genetic resemblance (S1 and S2 Fig). Figs 1 and 2 display the afHR of OP and fractures at common osteoporotic fracture sites among twins, full-siblings, half-siblings, and cousins. There is a clear relation to the degree of genetic resemblance in Figs 1 and 2. The Cumulative Incidence Function and log-log Survival Plots for OP and fractures at common osteoporotic fracture sites are presented in S3, S4, S5, S6 Figs, respectively. The log-log Plots indicates that there is no major violation of the proportional hazards assumption.

### Stratified analysis among full-siblings for hospital treated OP

Sex and age-stratified analysis were performed among full-siblings. In the sex-stratified analysis of familial HR for OP among full-siblings, the fully afHR was 2.37 (95%CI 2.20–2.56) for men and 2.19 (95%CI 2.11–2.28) for women (S5 Table).

In the age stratified analysis of familial HR for OP among full-siblings stratified by birth year (median year = 1976), the subjects born before 1976 (i.e., older subjects) had a fully afHR of 2.20 (95%CI 2.12–2.90) and for subjects born after 1976 (i.e., younger subjects) had a fully afHR of 22.88 (95%CI 13.98–37.45) (S6 Table).

### Stratified analysis among full-siblings for fractures at common osteoporotic fracture sites

Sex and age-stratified analysis were performed among full-siblings. In the sex-stratified analysis of familial HR for fractures at common osteoporotic fracture sites among full-siblings, the fully afHR was 1.44 (95%CI 1.42–1.46) for men and 1.44 (95%CI 1.42–1.46) for women (S5 Table).

In the age stratified analysis of familial HR for fractures at common osteoporotic fracture sites among full-siblings stratified by birth year (median year = 1976), the subjects born before 1976 (i.e., older subjects) had a fully afHR of 1.28 (95%CI 1.26–1.29) and for subjects born after 1976 (i.e., younger subjects) had a fully afHR of 1.74 (95%CI 1.70–1.78) (S6 Table).

### Concordant and discordant subanalysis

Concordant (same diagnosis in the relative pairs) and discordant analysis (different diagnosis in the relative pairs) were performed on twins, full-siblings, half-siblings and cousins, respectively, after deleting patients with M80 and M81 diagnosed simultaneously or M80 at first visit followed by M81. Fully afHRs are presented with 95%CI (S7 Table). Highest risks were observed for concordant diagnosis of M80, i.e., OP with fracture. The discordant analysis also showed that family history of OP increases risk of fractures at common osteoporotic fracture sites in relatives (S7 Table).

### Familial population attributable fraction and standardised incidence ratio

Familial population attributable fraction (PAF) and standardised incidence ratio (SIR) analysis were performed stratified by sex and birth year with 95% CI intervals for OP and fractures at common osteoporotic fracture sites, respectively. The analysis was performed for twins, full-siblings, half-siblings and cousins, respectively (S8 Table). The familial PAF for inheritance was highest for OP among twins (9%) (S8 Table).

### Additional analysis

In S9 and S10 Tables, the HRs for all the included covariates for OP and fractures at common osteoporotic fracture sites are added stepways and displayed in the twin models. Adjustment for birth year was the most important adjusting variable in S9 and S10 Tables.

In S11 Table, an aggregated analysis was performed with all familial relationships included in the same model, i.e., twins, full-siblings, half-siblings and cousins for both OP and fractures at common osteoporotic fracture sites comparing odds ratio (Logistic regression model), hazard ratio (Cox regression model), and competing-event analysis according to Fine and Gray. The results are similar even if all familial relationships are included in the same model. Moreover, the results are similar using logistic regression, Cox regression, and competing-event analysis.

In S12 and S13 Tables, we present familial HRs among only the oldest full-sibling couple for OP and fractures at common osteoporotic fracture sites, respectively, for double-entry (n = 4 602 892) (S12 Table) and single entry (n = 2 301 446) (S13 Table). This gives similar results.

In S14 Table, we present familial HRs for parent-offspring for OP and fractures at common osteoporotic fracture sites, respectively. All families with parents who died before 1997 were excluded. The fully afHR for OP was 2.23 (95%CI 2.14–2.32), and the fully afHR for fractures at common osteoporotic fracture sites was 1.27 (95%CI 1.26–1.28) (S14 Table), similar to the results among full siblings.

In S15 Table, we present familial HRs for full siblings for fractures at common osteoporotic fracture sites after the exclusion of cases with first event of radius fracture (ICD-10 S525). The fully afHR for fractures at common osteoporotic fracture sites was 1.35 (95%CI 1.32–1.37) when excluding radius fractures (S15 Table) compared to fully afHR of 1.44 (95%CI 1.43–1.46) when including radius fractures (Table 3).

### Familial hazard ratio for 8 different hospital treated common osteoporitc fracture sites

The familial risk among full-siblings was similar for S22, S32, S72, S422, S423, S525, S825, and S826 compared with inclusion of only five fracture sites (S22, S32, S422, S525, S72) with afHR of 1.38 (95%CI 1.36–1.39) in model 3 (S16 Table). Moreover, with inclusion only of those with fall-related fractures (ICD-10 codes W00-W19) the familial aHR among full-siblings was slightly higher 1.42 (1.41–1.44) in model 3 (S17 Table).

In S18 Table prescription data were examined among affected full siblings using the Swedish Prescribed Drug Register. Overall, 37,045 (68.2%) of 54,306 full siblings with an OP diagnosis (M80 or M81) had, at some point during the follow-up period (i.e., intention to treat), been prescribed medications affecting bone structure and mineralization (Anatomical Therapeutic Chemical [ATC] code M05B). Furthermore, 44,904 (82.7%) individuals had been prescribed either a combination of vitamin D and its analogues (ATC code A11CC) and calcium (ATC code A12A), or calcium in combination with vitamin D and/or other agents (ATC code A12AX) during follow-up.

## Discussion

This large real-world nationwide study presents estimates for familial risks in first-, second-, and third-degree relatives for hospital treated OP and fractures at common osteoporotic fracture sites. The high risk of hospital treated OP among young individuals might be related to rare variants [29] or enrichment of polygenic variants and a high PRS [30–33]. The higher afHR for OP and fractures at young age in the stratified analysis suggests that early-onset OP might be underestimated in the general population (Tables 4 and 5 and S6 Table) [29], though we cannot rule out the contribution of shared environment. In fact, a recent published study using whole genome sequencing shows that 20% of human phenotypes might be cause by rare variants [44]. The contribution rare and common variants to OP and fractures will be important to determine [29,44].

We identified hereditary components of both OP and fractures at common osteoporotic fracture sites, with higher heritability for OP than for fractures. The strongest aggregations of OP were observed in pairs of twins and full-siblings, i.e., first-degree relatives, whereas this association was attenuated among half-siblings and cousin pairs, i.e., second and third-degree relatives. This correlation between familial risk and genetic resemblance suggests a genetic contribution to OP [27], but cannot exclude an environmental contribution. Significant familial associations in third-degree relatives, i.e., cousins of affected patients support that genetic factors influence familial aggregation, since cousins usually not share household [27]. For the same reason, one can argue that full-siblings and twins share household, which however could not solely explain the higher familial risk among twins compared with full-siblings. Unfortunately, we have no information about the zygosity of the included twins. However, the average genetic resemblance for twins (0.66) is higher than for full-siblings (0.50) which might suggest a genetic explanation for the higher HR among twins than full-siblings. However, we cannot exclude the contribution of shared environment. Twins unlike full-siblings share intrauterine environment.

Half-siblings share 25% of their genetic material on average, whereas first cousins share only 12.5% of their genetic material. One would therefor think that half-siblings would have a stronger afHR for OP and fractures at common osteoporotic fracture sites than cousins. However, this was not the case, as illustrated in Fig 1 and 2. The reason for this is likely a direct register effect due to the fact that cousins are slightly younger with a mean age for the whole group of 29.95 years (S4 Table), compared to half-siblings with a mean age for the whole group of 36.48 years (S3 Table), and the younger the individual the stronger the familial risk will be over other risk factors such as age and other comorbidities.

In S12 and S13 Tables, we analysed fully afHRs for the two oldest full-siblings in a family to account for the fact that some families could have a larger cluster of OP or fractures at common osteoporotic fracture sites, respectively. The fully afHRs were nearly the same as using all siblings in all families (i.e., complete ascertainment of all families). Moreover, using double entry or single entry did not affect the results to any major degree. We also did a subanalysis of parent-offspring for OP and fractures at common osteoporotic fracture sites in S14 Table to investigate fully afHRs for OP and fractures at common osteoporotic fracture sites in parents and offspring. The fully afHRs were very similar to the fully afHRs for full-siblings.

The studied patients were all Swedish-born people with Swedish-born parents, born between 1932 and 2018, thus being between 0 and 87 years old, to be able to detect early-onset OP [29]. Still, it is known that bone loss usually occurs with advancing age, thus resulting in increased prevalence of OP with age [45]. Previous studies have estimated the prevalence of OP in Sweden to be 21.2% in women and 6.3% in men in the ages of 50–84 years [45]. We found the prevalence of specialist diagnosed OP to be 0.88% and the prevalence of fractures at common osteoporotic fracture sites to be 6.16% in the whole studied cohort (Table 1). Our lower prevalence findings are probably due to the inclusion of young subjects. This finding is more obvious when studying the groups separately. The youngest mean age was found in cousins with a mean age of 29.95 years among all subjects, 40.79 years in subjects with OP but only 22.54 years among subjects with OP fracture. The prevalence of OP in this group was 0.14% and for fractures at common osteoporotic fracture sites it was 4.48% (S4 Table). The highest mean age was found in full-siblings with a mean age of 42.43 years among all subjects, 64.82 years in subjects with OP and 43.94 years in subjects with fractures at common osteoporotic fracture sites. The prevalence of OP was 0.95% and for fractures at common osteoporotic fracture sites it was 6.34% in this group (S2 Table). Higher prevalence in older patients in our material correlates well with previous studies [45].

The most important OP risk factors to adjust for are age and sex, as shown in this study. In S9 Table and S10 Table, we clearly show that adjusting for age (Model B) is the most important adjustment, followed by adjusting for sex (Model C) in familial HRs for twins in OP and OP fractures, respectively. The mean age in the OP group was 64.38 years and in the fractures at common osteoporotic fracture sites group was 42.78 years, compared to 41.16 years in the whole cohort. That the mean age is lower in the fractures at common osteoporotic fracture sites group is likely due to that we included distal radius fractures (ICD-10 S525) in the study, and that this fracture is the most common fracture globally, both common in children and elderly [46]. Patients with high energy trauma might have been included. In a study of fractures in children in Sweden, it was shown that up to 25% of fractures involve the distal end of the radius [47]. However, most distal radius fractures occur in the adult population due to underlying OP [48], which makes it important to include distal radius fractures in this study. The mean age of radius fracture in southern Sweden was shown in one previous study from 2020 to be around 66 years [49], compared to the mean age of hip fractures that was 81.9 years in Sweden in a study from 2017 [50]. In S15 Table, we excluded radius fractures but the difference was only minor; fully afHR of 1.35 (95%CI 1.32–1.37) when excluding radius fractures compared to fully afHR of 1.44 (95%CI 1.43–1.46) when including radius fractures, suggesting that the familiar risk was only slightly lower when excluding this common fracture (S15 Table and Table 3). This is in line with the age-stratified analysis showing highest afHR in young individuals born from 1977 to 2018 for OP and for fractures. Moreover, the discordant analysis showed that family history of OP increases the risk of fractures at common osteoporotic fracture sites, suggesting that subclinical OP at young age in some cases due to early-onset monogenic or polygenic disease [29].

There were 82.61% females in the OP group and 50.25% females in the fractures at common osteoporotic fracture sites group, compared to 48.77% females in the whole cohort. OP is more common in women [2], but when it comes to fractures at common osteoporotic fracture sites, it seems to be equally distributed between males and females in this study. This is not consistent with previous studies; men accounted for 29% of all fractures associated with OP in an American study [51]. This could be explained by the inclusion of non-OP related fractures due to high energy trauma, in the fractures at common osteoporotic fracture sites group. However, as mentioned above, family history of OP is associated with fractures at common osteoporotic fracture sites, especially among young individuals.

In the stratified analysis of gender (S5 Table), the afHR was slightly higher in males; fully afHR of 2.37 (95%CI 2.20–2.56) for OP in full-siblings in the fully adjusted model compared to a fully afHR of 2.19 (95%CI 2.11–2.28) in women. This shows that genetics may play a stronger part in OP in men than in women but are an especially strong risk factor in younger individuals, both men and premenopausal women. This is the so-called Carter effect, i.e., the prediction that in a complex genetic condition with different prevalence by sex, familial recurrence will be higher when an affected family member is of the less commonly affected sex [52]. However, in OP related fractures, it was nearly the same fully afHR of 1.44 (95%CI 1.42–1.46).

Finally, we did an analysis in S8 Table to check for the familial PAF and the SIR. For example, the PAF for twins was 9% (95% CI 7–10) and the SIR was 3.65 (95%CI 3.15–4.14). This pattern suggests that whilst direct genetic inheritance is important, other factors such as environmental, epigenetic and familial lifestyle factors play a significant role in OP risk.

### Strength and limitations

In common with all epidemiological studies, the interpretation of results is constrained by time (1997–2018) and geographical location (Sweden), resulting in some bias in time period and location. Many of the limitations in the study involve a nondifferential bias with regards to familial risks, for instance the lack of primary care data. A limitation is the lack of information about menopause, which cannot be accounted for. Age of menopause has been suggested to be partially genetic controlled, and could thus contribute to the observed familial associations among females, but not males, that was similar increased with overlapping confidence intervals (S5 Table). Another limitation is that primary care data were not included. However, it has been reported that in Sweden only 1 out of 5 patients were followed up in primary care after their first fragility fracture [53]. A strength is that both specialist treated outpatients and inpatients, i.e., hospital treated cases, were included. Moreover, just over 5% received bone-specific drugs within 6 months in primary care [53]. Adding primary care data is therefore unlikely to be beneficial. However, it is likely that the prevalence of OP in Sweden is higher than suggested in this study, since OP is a low-status disease, meaning it is often ignored and undiagnosed in favour of more high-status diseases such as life-threatening diseases and diseases that warrant expensive treatments. Severe or early onset cases are often enriched for high penetrance variants and are therefore particularly informative for genetic studies [54]. The present study mainly reflects genetics of more severe specialist treated cases.

The hospital treated OP diagnosis came from the NPR, which is a reliable register, since it included both hospital discharge diagnoses from 1997 onwards, as well as specialist outpatient clinic diagnoses from 2001 onwards [38,39]. The diagnosis of OP is therefore expected to be correct, especially as bone density measurement has been used since long time for diagnosis of OP in Sweden [55]. It is therefore likely that included patients had OP of later stages, when they needed to be referred from primary health care to specialised care at hospitals or specialist clinics. Another limitation to consider is that we do not have any information whether the OP diagnoses of the subjects in this cohort were confirmed with BMD or not. The prevalence of hospital treated fractures is, however, expected to be a fairly accurate estimate as most fractures are seen in specialised health care in Sweden (i.e., emergency clinics at hospitals) with high completeness though the true number of fractures might be overestimated in NPR [56].

Lifestyle factors such as physical activity are important to consider when discussing risk factors for OP and fractures at common osteoporotic fracture sites, and this could partially be adjusted for by adjusting for low or high education level. Thus, residual confounding is therefore likely to exist for lifestyle factors. However, we adjusted for relevant comorbidities in the used registers. We could not adjust for smoking as this diagnose is rarely found in the registers. However, COPD could serve as a proxy for heavy smoking. Still, residual confounding for smoking is likely to exist. The models adjust for several comorbid conditions (COPD, alcohol use disorder, RA, PM and GCA, IBD, and asthma), which are themselves partially heritable and may act as intermediates on the causal pathway between genetic susceptibility and OP. Consequently, such adjustment may lead to overadjustment, potentially attenuating the estimated genetically mediated effects.

However, adjustments for comorbidities had little influence of the familial risks and it is unlikely that adjustment for more comorbidities, for instance chronic kidney disease in the present relatively young cohort will affect the familial risk to any major degree (S9 and S10 Tables).

In conclusion, the present nationwide family study confirms that there is a hereditary pattern for hospital treated OP and fractures. Family history is associated with hospital treated OP and fractures in the general population in Sweden. Information about familial clustering of hospital treated OP and fracture may be of importance for identification of high risk families.

## Supporting information

S1 Table Characteristics of twin patients with osteoporosis (OP) or fractures at common osteoporotic fracture sites (=fractures at OP sites) compared with their non-affected twin relatives (unique individuals). (DOCX)

S2 Table Characteristics of full sibling patients with osteoporosis (OP) or fractures at common osteoporotic fracture sites (=fractures at OP sites) compared with their non-affected full sibling relatives (unique individuals). (DOCX)

S3 Table Characteristics of half-sibling patients with osteoporosis (OP) or fractures at common osteoporotic fracture sites (=Fractures at OP sites) compared with their non-affected half-sibling relatives (unique individuals). (DOCX)

S4 Table Characteristics of cousin patients with osteoporosis (OP) or common osteoporotic fracture sites (=Fractures at OP sites) compared with their non-affected cousin relatives (unique individuals). (DOCX)

S5 Table Hazard ratios (HRs) with 95% confidence intervals (95% CIs) for patients with osteoporosis (OP) or fractures at common osteoporotic fracture sites (=Fractures at OP sites) compared with their non-affected relatives among full siblings, stratified by sex. (DOCX)

S6 Table Hazard ratios (HRs) with 95% confidence intervals (95% CIs) for patients with osteoporosis (OP) or fractures at common osteoporotic fracture sites (=Fractures at OP sites) compared with their non-affected relatives among full siblings, stratified by birth year. (DOCX)

S7 Table Discordance analysis (after deleting patients with M80 and M81 diagnosed simultaneously or M80 at first visit followed by M81), full models. Familial Hazard ratios (HRs) with 95% confidence intervals (95% CI) for patients with osteoporosis (OP) or fractures at common osteoporotic fracture sites (=Fractures at OP sites).
(DOCX)

S8 Table Familial population attributable fraction (PAF) and familial Standardised incidence ratio (SIR) with 95% confidence intervals (95% CI) for osteoporosis (OP) and fractures at common osteoporotic fracture sites (=Fractures at OP sites). (DOCX)

S9 Table Familial Hazard ratios (HRs) with 95% confidence intervals (95% CI) among twins for osteoporosis (OP) (ICD-10 = M80, M81) for individuals with relative history of osteoporosis compared with those with non-affected relatives (n = 149002). HRs for comorbidities are displayed. (DOCX)

S10 Table Familial Hazard ratios (HRs) with 95% confidence intervals (95% CI) among twins for osteoporosis-related fractures (ICD-10 = S22, S32, S422, S525, S72) for individuals with relative history of osteoporosis fracture compared with those with non-affected relatives (n = 149002). HRs for comorbidities are displayed. (DOCX)

S11 Table Aggregated analysis with all familial relationships included in the same model. Fully adjusted Odds ratio, Hazard ratio and Competing-event analysis according to Fine and Gray in family members by degree of relationship (siblings, half-siblings, and cousins in one dataset for osteoporosis (ICD-10 = M80, M81), fractures at common osteoporotic fracture sites (=Fractures at OP sites) (ICD-10 = S22, S32, S422, S525, S72) for individuals with relative history of osteoporosis or Fractures at OP sites compared with those with non-affected relatives. (DOCX)

S12 Table Familial Hazard ratios (HRs) with 95% confidence intervals (95% CI) with inclusion of only the two oldest full-siblings (double entry) in a pair for osteoporosis (ICD-10 = M80,M81) or fractures at common osteoporotic fracture sites (=Fractures at OP sites) (ICD-10 = S22, S32, S422, S525, S72) for individuals with relative history of osteoporosis or fracture at OP sites compared with those with non-affected relatives. n = 4602892. (DOCX)

S13 Table Familial Hazard ratios (HRs) with 95% confidence intervals (95% CI) with inclusion of only the two oldest full-siblings (single entry) for osteoporosis (ICD-10 = M80,M81), fractures at common osteoporotic fracture sites (=Fractures at OP sites)(ICD-10 = S22, S32, S422, S525, S72) for individuals with relative history of osteoporosis or fractures at OP sites compared with those with non-affected relatives. n = 2301446. (DOCX)

S14 Table Familial Hazard ratios (HRs) with 95% confidence intervals (95% CI) among parent-offspring for osteoporosis (OP) (ICD-10 = M80,M81) and fractures at common osteoporotic fracture sites (=fractures at OP sites) (ICD-10 = S22, S32, S422, S525, S72) for individuals with parent history of OP or fractures at OP sites compared with those with non-affected relatives. All families with parents who died before 1997 were excluded. n = 5133637. (DOCX)

S15 Table Familial Hazard ratios (HRs) with 95% confidence intervals (95% CI) among full-siblings for fractures at common osteoporotic fracture sites (=fractures at OP sites) (ICD-10 = S22, S32, S422, S72) (after exclusion of individuals diagnosed with first fracture of distal radius S525) for individuals with relative history of fractures at OP sites compared with those with non-affected relatives. (n = 9 447 472). (DOCX)

S16 Table Hazard ratios (HRs) with 95% confidence intervals (95% CIs) among full-sublings for patients with fractures at common osteoporotic fracture sites (=fractures at OP sites, i.e., ICD-10 codes S22, S32, S72, S422, S423, S525, S825, and S826) compared with their non-affected relatives. (DOCX)

S17 Table Hazard ratios (HRs) with 95% confidence intervals (95% CIs) among full-sublings for patients with fractures at common osteoporotic fracture sites (=fractures at OP sites S22, S32, S72, S422, S423, S525, S825, and S826) compared with their non-affected relatives. Only patient with ICD-10 codes W00-W19 were included, i.e., fall related fractures (Slipping, tripping, stumbling and falls). (DOCX)

S18 Table Distribution of treatment groups among affected full siblings using the Swedish Prescribed Drug Register with osteoporosis with fracture (M80) or osteoporosis without fracture (M81), and combined M80/M81 diagnoses. (DOCX)

S1 Fig Kaplan-Meier osteoporosis (OP) free survival estimates by familial history among twins, full-siblings, half-siblings and cousins for individuals with relative history of osteoporosis compared with those with non-affected relatives. (DOCX)

S2 Fig Kaplan-Meier fractures at common osteoporotic fracture sites (=fractures at OP sites) free survival estimates by familial history among twins, full-siblings, half-siblings and cousins for individuals with relative history of osteoporosis fracture compared with those with non-affected relatives. (DOCX)

S3 Fig Cumulative Incidence Function (CIF) of osteoporosis (OP) estimates by familial history among twins, full-siblings, half-siblings and cousins for individuals with relative history of osteoporosis compared with those with non-affected relatives. (DOCX)

S4 Fig Cumulative Incidence Function (CIF) of fractures at common osteoporotic fracture sites (=fractures at OP sites) estimates by familial history among twins, full-siblings, half-siblings and cousins for individuals with relative history of fractures at OP sites compared with those with non-affected relatives. (DOCX)

S5 Fig Complementary log-log Survival Plot of osteoporosis (OP) estimates by familial history among twins, full-siblings, half-siblings and cousins for individuals with relative history of osteoporosis compared with those with non-affected relatives. (DOCX)

S6 Fig Complementary log-log Survival Plot of fractures at common osteoporotic fracture sites (=fractures at OP sites) estimates by familial history among twins, full-siblings, half-siblings and cousins for individuals with relative history of fractures at OP sites compared with those with non-affected relatives. (DOCX)
