# Supplementary material for: Familial risks of hospital treated osteoporosis and fractures in first-, second- and third-degree relatives - A Swedish nationwide real-world family study
Source: PLoS One. 2026 Sep 11;21(9):e0358074. doi: 10.1371/journal.pone.0358074 (PMC13567828; doi:10.1371/journal.pone.0358074)
Supplement: Supplementary file 1 [file pone.0358074.s001.docx]

| **Supplementary Table 1. Characteristics of twin patients with osteoporosis (OP) or fractures at common osteoporotic fracture sites (=fractures at OP sites) compared with their non-affected twin relatives (unique individuals).** | | | | | | | |
| --- | --- | --- | --- | --- | --- | --- | --- |
|  | **All individuals**    n= 146462 (100%) | **No osteoporosis**  n = 145001 (99.00) | **Osteoporosis**  n = 1461 (1.00) | ***P-value*** | **No-Fractures at OP sites**  n = 137324 (93.76) | **Fractures at OP sites**  n = 9138 (6.24) | ***P-value*** |
| **Sex**^£^ |  |  |  |  |  |  |  |
| Male | 73308 (50.05) | 73065 (50.39) | 243  (16.63) | <0.001 | 68972 (50.23) | 4336  (47.45) | <0.001 |
| Female | 73154 (49.95) | 71936 (49.61) | 1218  (83.37) |  | 68352 (49.77) | 4802 (52.55) |  |
| **Education**^£^ |  |  |  |  |  |  |  |
| > 11y | 70967 (48.45) | 70317 (48.49) | 388 (26.56) | <0.001 | 67423 (49.10) | 3585 (39.23) | <0.001 |
| **Year of birth*** |  |  |  |  |  |  |  |
| Mean (SD) | 1980.17 (24.44) | 1980.52 (24.30) | 1946.01  (11.14) | <0.001 | 1981.06 (24.23) | 1966.80  (23.82) | <0.001 |
| Median (IQR) | 1984  (1958-2001) | 1985  (1959-2002) | 1944  (1939-1950) |  | 1985  (1960-2002) | 1961  (1945-1992) |  |
| (Range) | (1932-2018) | (1932-2018) | (1932-2012) |  | (1932-2018) | (1932-2017) |  |
| **Age at end follow-up*** |  |  |  |  |  |  |  |
| Mean (SD) | 37.52  (23.92) | 37.51  (23.91) | 65.22  (12.09) | <0.001 | 36.97  (23.84) | 41.59  (24.96) | <0.001 |
| Median (IQR) | 33.72  (16.67-59.00) | 33.24  (16.91-58.75) | 67.09  (60.02-73.15) |  | 32.91  (16.25-58.00) | 48.46  (14.05-63.68) |  |
| (Range) | (0.00-87.00) | (0.00-87.00) | (0.40-85.45) |  | (0.00-87.00) | (0.06-85.97) |  |
| **Comorbidities**^£^**; %** |  |  |  |  |  |  |  |
| Obesity | 1.88 | 1.86 | 3.29 | <0.001 | 1.82 | 2.78 | <0.001 |
| COPD | 1.70 | 1.54 | 17.18 | <0.001 | 1.42 | 5.81 | <0.001 |
| Alcohol use disorder | 2.93 | 2.90 | 5.61 | <0.001 | 2.60 | 7.87 | <0.001 |
| RA | 0.80 | 0.71 | 9.10 | <0.001 | 0.71 | 2.07 | <0.001 |
| PMR and GCA | 0.42 | 0.36 | 6.57 | <0.001 | 0.37 | 1.18 | <0.001 |
| IBD | 0.96 | 0.94 | 3.63 | <0.001 | 0.92 | 1.61 | <0.001 |
| Asthma | 7.96 | 7.96 | 7.94 | 0.980 | 7.94 | 8.19 | 0.406 |
| **Exposure (number affected)** |  |  |  |  |  |  |  |
|  | **Twins** | 1290  (0.89) | 178  (12.18) |  | 8002  (5.89) | 1252  (13.70) |  |
|  | **Triplets** | --- | --- |  | 15  (0.01) | 0  (0.00) |  |
| Age at end of follow-up: death, migration, osteoporosis, osteoporosis fracture diagnosis or end of study period on December 31, 2018, whichever came first; SD, standard deviation; IQR, interquartile range.  **P-value*: Mann-Whitney test.  ^£^*P-value*: Chi-square test.  Abbreviations: chronic obstructive pulmonary disease (COPD), rheumatoid arthritis (RA), polymyalgia rheumatica (PMR) giant cell arthritis (GCA),, and inflammatory bowel disease (IBD). | | | | | | | |
