# Supplementary material for: Familial risks of hospital treated osteoporosis and fractures in first-, second- and third-degree relatives - A Swedish nationwide real-world family study
Source: PLoS One. 2026 Sep 11;21(9):e0358074. doi: 10.1371/journal.pone.0358074 (PMC13567828; doi:10.1371/journal.pone.0358074)
Supplement: Supplementary file 2 [file pone.0358074.s002.docx]

| **Supplementary Table 2. Characteristics of full sibling patients with osteoporosis (OP) or fractures at common osteoporotic fracture sites (=fractures at OP sites) compared with their non-affected full sibling relatives (unique individuals).** | | | | | | | |
| --- | --- | --- | --- | --- | --- | --- | --- |
|  | **All individuals**    n= 5714784 (100%) | **No osteoporosis**  n = 5660478 (99.05) | **Osteoporosis**  n = 54306 (0.95) | ***P-value*** | **No-Fractures at OP sites**  n = 5352414 (93.66) | **Fractures at OP sites**  n = 362370 (6.34) | ***P-value*** |
| **Sex**^£^ |  |  |  |  |  |  |  |
| Male | 2930213 (51.27) | 2920884 (51.60) | 9329 (17.18) | <0.001 | 2751649 (51.41) | 178564  (49.28) | <0.001 |
| Female | 2784571 (48.73) | 2739594 (48.40) | 44977  (82.82) |  | 2600765 (48.59) | 183806 (50.72) |  |
| **Education**^£^ |  |  |  |  |  |  |  |
| > 11y | 2657612 (46.50) | 2632017 (46.50) | 15839 (29.17) | <0.001 | 2518397 (47.05) | 140371 (38.74) | <0.001 |
| **Year of birth*** |  |  |  |  |  |  |  |
| Mean (SD) | 1975.23 (23.22) | 1975.51 (23.13) | 1946.38  (11.13) | <0.001 | 1975.96 (23.09) | 1964.45  (22.45) | <0.001 |
| Median (IQR) | 1974  (1955-1994) | 1974  (1956-1994) | 1944  (1939-1951) |  | 1975  (1957-1995) | 1958  (1945-1988) |  |
| (Range) | (1932-2018) | (1932-2018) | (1932-2014) |  | (1932-2018) | (1932-2018) |  |
| **Age at end follow-up*** |  |  |  |  |  |  |  |
| Mean (SD) | 42.43  (22.71) | 42.49  (22.72) | 64.82  (12.00) | <0.001 | 42.04  (22.68) | 43.94  (23.72) | <0.001 |
| Median (IQR) | 43.84  (23.62-61.75) | 43.67  (24.00-61.75) | 66.72  (59.41-72.94) |  | 43.16  (23.58-61.16) | 50.23  (17.94-63.97) |  |
| (Range) | (0.00-87.00) | (0.00-87.00) | (0.20-86.71) |  | (0.00-87.00) | (0.02-86.90) |  |
| **Comorbidities**^£^**; %** |  |  |  |  |  |  |  |
| Obesity | 2.85 | 2.84 | 3.73 | <0.001 | 2.80 | 3.61 | <0.001 |
| COPD | 1.77 | 1.64 | 16.07 | <0.001 | 1.51 | 5.69 | <0.001 |
| Alcohol use disorder | 3.04 | 3.02 | 5.28 | <0.001 | 2.71 | 7.84 | <0.001 |
| RA | 0.90 | 0.82 | 9.79 | <0.001 | 0.82 | 2.11 | <0.001 |
| PMR and GCA | 0.47 | 0.41 | 5.95 | <0.001 | 0.41 | 1.24 | <0.001 |
| IBD | 1.18 | 1.15 | 4.08 | <0.001 | 1.15 | 1.63 | <0.001 |
| Asthma | 6.25 | 6.22 | 10.09 | <0.001 | 6.17 | 7.48 | <0.001 |
| **Exposure (number affected)** | |  |  |  |  |  |  |
|  | **1** | 94514  (1.67) | 6102  (11.24) |  | 507341  (9.48) | 60190  (16.61) |  |
|  | **2** | 5053  (0.09) | 584  (1.08) |  | 41631  (0.78) | 7444  (2.05) |  |
|  | **3** | 377  (0.01) | 86  (0.16) |  | 5089  (0.10) | 1130  (0.31) |  |
|  | **4** | 45  (0.00) | 10  (0.02) |  | 682  (0.01) | 175  (0.05) |  |
|  | **5** | 1  (0.00) | 0  (0.00) |  | 88  (0.00) | 28  (0.01) |  |
|  | **6** | --- | --- |  | 13  (0.00) | 0  (0.00) |  |
| Age at end of follow-up: death, migration, osteoporosis, osteoporosis fracture diagnosis or end of study period on December 31, 2018, whichever came first; SD, standard deviation; IQR, interquartile range.  **P-value*: Mann-Whitney test.  ^£^*P-value*: Chi-square test.  Abbreviations: chronic obstructive pulmonary disease (COPD), rheumatoid arthritis (RA), polymyalgia rheumatica (PMR), giant cell arthritis (GCA), and inflammatory bowel disease (IBD). | | | | | | | |
