# Supplementary material for: Familial risks of hospital treated osteoporosis and fractures in first-, second- and third-degree relatives - A Swedish nationwide real-world family study
Source: PLoS One. 2026 Sep 11;21(9):e0358074. doi: 10.1371/journal.pone.0358074 (PMC13567828; doi:10.1371/journal.pone.0358074)
Supplement: Supplementary file 3 [file pone.0358074.s003.docx]

| **Supplementary Table 3. Characteristics of half-sibling patients with osteoporosis (OP) or fractures at common osteoporotic fracture sites (=Fractures at OP sites) compared with their non-affected half-sibling relatives (unique individuals).** | | | | | | | |
| --- | --- | --- | --- | --- | --- | --- | --- |
|  | **All individuals**    n= 1375039 (100%) | **No osteoporosis**  n = 1368352 (99.51) | **Osteoporosis**  n = 6687 (0.49) | ***P-value*** | **No-Fractures at OP sites**  n = 1294478 (94.14) | **Fractures at OP sites**  n = 80561 (5.86) | ***P-value*** |
| **Sex**^£^ |  |  |  |  |  |  |  |
| Male | 702062 (51.06) | 700719 (51.21) | 1343 (20.08) | <0.001 | 658109 (50.84) | 43953 (54.56) | <0.001 |
| Female | 672977 (48.94) | 667633 (48.79) | 5344  (79.92) |  | 636369 (49.16) | 36608 (45.44) |  |
| **Education**^£^ |  |  |  |  |  |  |  |
| > 11y | 500624 (36.41) | 495764 (36.23) | 1717 (25.68) | <0.001 | 474098 (36.62) | 27095 (33.63) | <0.001 |
| **Year of birth*** |  |  |  |  |  |  |  |
| Mean (SD) | 1981.34 (19.76) | 1981.48 (19.68) | 1953.29  (13.76) | <0.001 | 1981.75 (19.68) | 1974.83  (19.90) | <0.001 |
| Median (IQR) | 1982  (1966-1997) | 1983  (1966-1997) | 1951  (1944-1960) |  | 1983  (1967-1997) | 1977  (1958-1992) |  |
| (Range) | (1932-2018) | (1932-2018) | (1932-2014) |  | (1932-2018) | (1932-2018) |  |
| **Age at end follow-up*** |  |  |  |  |  |  |  |
| Mean (SD) | 36.48  (19.43) | 36.65  (19.40) | 58.05  (14.51) | <0.001 | 36.38  (19.40) | 33.21  (21.14) | <0.001 |
| Median (IQR) | 35.33  (21.08-51.75) | 35.42  (21.75-51.84) | 60.59  (51.59-68.02) |  | 35.33  (21.33-51.58) | 30.41  (13.27-51.66) |  |
| (Range) | (0.00-87.00) | (0.00-87.00) | (0.20-85.86) |  | (0.00-87.00) | (0.04-86.75) |  |
| **Comorbidities**^£^**; %** |  |  |  |  |  |  |  |
| Obesity | 4.10 | 4.09 | 5.83 | <0.001 | 4.04 | 4.93 | <0.001 |
| COPD | 1.25 | 1.17 | 17.74 | <0.001 | 1.07 | 4.17 | <0.001 |
| Alcohol use disorder | 4.79 | 4.77 | 8.40 | <0.001 | 4.37 | 11.52 | <0.001 |
| RA | 0.67 | 0.62 | 9.00 | <0.001 | 0.63 | 1.29 | <0.001 |
| PMR and GCA | 0.20 | 0.18 | 4.04 | <0.001 | 0.18 | 0.48 | <0.001 |
| IBD | 1.15 | 1.13 | 5.49 | <0.001 | 1.13 | 1.55 | <0.001 |
| Asthma | 7.66 | 7.64 | 12.25 | <0.001 | 7.57 | 9.17 | <0.001 |
| **Exposure (number affected)** | |  |  |  |  |  |  |
|  | **1** | 12811  (0.94) | 355  (5.31) |  | 134531  (10.39) | 10244  (12.72) |  |
|  | **2** | 396  (0.03) | 25  (0.37) |  | 10097  (0.78) | 858  (1.07) |  |
|  | **3** | 27  (0.00) | 1  (0.01) |  | 828  (0.06) | 94  (0.12) |  |
|  | **4** | 1  (0.00) | 0  (0.00) |  | 86  (0.01) | 13  (0.02) |  |
|  | **5** | --- | --- |  | 9  (0.00) | 0  (0.00) |  |
|  | **6** | --- | --- |  | 1  (0.00) | 0  (0.00) |  |
| Age at end of follow-up: death, migration, osteoporosis, osteoporosis fracture diagnosis or end of study period on December 31, 2018, whichever came first; SD, standard deviation; IQR, interquartile range.  **P-value*: Mann-Whitney test.  ^£^*P-value*: Chi-square test.  Abbreviations: chronic obstructive pulmonary disease (COPD), rheumatoid arthritis (RA), polymyalgia rheumatica (PMR) giant cell arthritis (GCA),, and inflammatory bowel disease (IBD). | | | | | | | |
