# Supplementary material for: Familial risks of hospital treated osteoporosis and fractures in first-, second- and third-degree relatives - A Swedish nationwide real-world family study
Source: PLoS One. 2026 Sep 11;21(9):e0358074. doi: 10.1371/journal.pone.0358074 (PMC13567828; doi:10.1371/journal.pone.0358074)
Supplement: Supplementary file 4 [file pone.0358074.s004.docx]

| **Supplementary Table 4. Characteristics of cousin patients with osteoporosis (OP) or common osteoporotic fracture sites (=Fractures at OP sites) compared with their non-affected cousin relatives (unique individuals).** | | | | | | | |
| --- | --- | --- | --- | --- | --- | --- | --- |
|  | **All individuals**    n= 4149511 (100%) | **No osteoporosis**  n = 4143705 (99.86) | **Osteoporosis**  n = 5806 (0.14) | ***P-value*** | **No-Fractures at OP sites**  n = 3963733 (95.52) | **Fractures at OP sites**  n = 185778 (4.48) | ***P-value*** |
| **Sex**^£^ |  |  |  |  |  |  |  |
| Male | 2131882 (51.38) | 2130144 (51.41) | 1738 (29.93) | <0.001 | 2023290 (51.05) | 108592 (58.45) | <0.001 |
| Female | 2017629 (48.62) | 2013561 (48.59) | 4068  (70.07) |  | 1940443 (48.95) | 77186 (41.55) |  |
| **Education**^£^ |  |  |  |  |  |  |  |
| > 11y | 2 207 738 (53.20) | 2194754 (52.97) | 2502 (43.09) | <0.001 | 2113561 (53.32) | 95476 (51.39) | <0.001 |
| **Year of birth*** |  |  |  |  |  |  |  |
| Mean (SD) | 1988.14 (16.66) | 1988.16 (16.66) | 1970.70  (12.46) | <0.001 | 1988.28 (16.78) | 1985.08  (13.65) | <0.001 |
| Median (IQR) | 1988  (1974-2002) | 1988  (1974-2002) | 1968  (1961-1978) |  | 1988  (1974-2003) | 1989  (1974-1995) |  |
| (Range) | (1947-2018) | (1947-2018) | (1948-2016) |  | (1948-2018) | (1947-2018) |  |
| **Age at end follow-up*** |  |  |  |  |  |  |  |
| Mean (SD) | 29.95  (16.58) | 30.17  (16.57) | 40.79  (13.67) | <0.001 | 30.05  (16.69) | 22.54  (14.69) | <0.001 |
| Median (IQR) | 29.75  (15.91-43.91) | 29.91  (16.33-44.08) | 42.97  (31.49-51.81) |  | 30.00  (15.75-44.08) | 16.63  (11.22-34.06) |  |
| (Range) | (0.00-72) | (0.00-71.58) | (0.20-66.54) |  | (0.00-71.00) | (0.02-69.12) |  |
| **Comorbidities**^£^**; %** |  |  |  |  |  |  |  |
| Obesity | 2.85 | 2.85 | 5.60 | <0.001 | 2.81 | 3.71 | <0.001 |
| COPD | 0.26 | 0.26 | 5.91 | <0.001 | 0.24 | 0.73 | <0.001 |
| Alcohol use disorder | 2.71 | 2.70 | 6.60 | <0.001 | 2.52 | 6.69 | <0.001 |
| RA | 0.36 | 0.35 | 8.27 | <0.001 | 0.35 | 0.54 | <0.001 |
| PMR and GCA | 0.03 | 0.03 | 0.88 | <0.001 | 0.03 | 0.07 | <0.001 |
| IBD | 0.98 | 0.97 | 8.20 | <0.001 | 0.96 | 1.28 | <0.001 |
| Asthma | 7.42 | 7.41 | 11.73 | <0.001 | 7.35 | 8.96 | <0.001 |
| **Exposure (number affected)** | |  |  |  |  |  |  |
|  | **1** | 35657  (0.86) | 160  (2.76) |  | 720196  (18.17) | 40761  (21.94) |  |
|  | **2** | 1094  (0.03) | 13  (0.22) |  | 149626  (3.77) | 9237  (4.97) |  |
|  | **3** | 80  (0.00) | 0  (0.00) |  | 28893  (0.73) | 1949  (1.05) |  |
|  | **4** | 3  (0.00) | 0  (0.00) |  | 5888  (0.15) | 466  (0.25) |  |
|  | **5** | --- | --- |  | 1434  (0.04) | 96  (0.05) |  |
|  | **6** | --- | --- |  | 375  (0.01) | 25  (0.01) |  |
|  | **7** | --- | --- |  | 98  (0.00) | 10  (0.01) |  |
|  | **8** | --- | --- |  | 29  (0.00) | 1  (0.00) |  |
|  | **9** | --- | --- |  | 14  (0.00) | 1  (0.00) |  |
|  | **10** | --- | --- |  | 3  (0.00) | 0  (0.00) |  |
| Age at end of follow-up: death, migration, osteoporosis, osteoporosis fracture diagnosis or end of study period on December 31, 2018, whichever came first; SD, standard deviation; IQR, interquartile range.  **P-value*: Mann-Whitney test.  ^£^*P-value*: Chi-square test.  Abbreviations: chronic obstructive pulmonary disease (COPD), rheumatoid arthritis (RA), polymyalgia rheumatica (PMR), giant cell arthritis (GCA), and inflammatory bowel disease (IBD). | | | | | | | |
