# Supplementary material for: Familial risks of hospital treated osteoporosis and fractures in first-, second- and third-degree relatives - A Swedish nationwide real-world family study
Source: PLoS One. 2026 Sep 11;21(9):e0358074. doi: 10.1371/journal.pone.0358074 (PMC13567828; doi:10.1371/journal.pone.0358074)
Supplement: Supplementary file 6 [file pone.0358074.s006.docx]

| **Supplementary Table 6. Hazard ratios (HRs) with 95% confidence intervals (95% CIs) for patients with osteoporosis (OP) or fractures at common osteoporotic fracture sites (=Fractures at OP sites) compared with their non-affected relatives among full siblings, stratified by birth year.** | | | | | | | |
| --- | --- | --- | --- | --- | --- | --- | --- |
| Variable | Person-years,  No. | Cases, No./Persons at risk, No. | Incidence rate, cases/1000 person-years | Incidence rate ratio (95%CI) | HR(95% CI) | | |
|  |  |  |  |  | **Model 1** | **Model 2** | **Model 3** |
| **Birth y ≤ 1976** (n= 5878537) |  |  |  |  |  |  |  |
| Non-osteoporosis | 120377089 | 103718/5767433 | 0.86  (0.86-0.87) | 1 [Reference] | 1 [Reference] | 1 [Reference] | 1 [Reference] |
| Osteoporosis | 2207103 | 7520/ 111104 | 3.41  (3.33-3.49) | **3.95*****  (3.86-4.05) | **3.87*****  (3.73-4.02) | **2.30*****  (2.22-2.39) | **2.20*****  (2.12-2.9) |
| **Birth y > 1976** (n = 4112973) |  |  |  |  |  |  |  |
| Non-osteoporosis | 71098366 | 2218/4110573 | 0.03  (0.03-0.03) | 1 [Reference] | 1 [Reference] | 1 [Reference] | 1 [Reference] |
| Osteoporosis | 49258 | 48 /2400 | 0.97  (0.73-1.29) | **31.24*****  (23.47-41.58) | **28.87*****  (17.92-46.51) | **24.31*****  (15.05-39.27) | **22.88*****  (13.98-37.45) |
| **Birth y ≤ 1976** (n= 5878537) |  |  |  |  |  |  |  |
| Non-Fractures at OP sites | 109304030 | 435956/5379666 | 3.99  (3.98-4.00) | 1 [Reference] | 1 [Reference] | 1 [Reference] | 1 [Reference] |
| Fractures at OP sites | 9757941 | 62616/498871 | 6.42  (6.37-6.47) | **1.61*****  (1.59-1.62) | **1.59*****  (1.57-1.61) | **1.29*****  (1.28-1.31) | **1.28*****  (1.26-1.29) |
| **Birth y > 1976**(n = 4112973) |  |  |  |  |  |  |  |
| Non-Fractures at OP sites | 65384567 | 173160/3923420 | 2.65  (2.64-2.66) | 1 [Reference] | 1 [Reference] | 1 [Reference] | 1 [Reference] |
| Fractures at OP sites | 3623285 | 16692/189553 | 4.61  (4.54-4.68) | **1.74*****  (1.71-1.77) | **1.74*****  (1.70-1.78) | **1.76*****  (1.72-1.80) | **1.74*****  (1.70-1.78) |
| HR, Hazard ratio. Model 1 crude. Model 2 adjusted for sex, education and birth year. Model 3 adjusted additionally for obesity, chronic obstructive pulmonary disease (COPD), alcohol use disorder, rheumatoid arthritis (RA), polymyalgia rheumatica (PMR), giant cell arthritis (GCA), inflammatory bowel disease (IBD), and asthma. All calculations were based on double entry. Significance levels: * p<0.05, ** p<0.01, *** p<0.001 | | | | | | | |
