# Supplementary material for: Familial risks of hospital treated osteoporosis and fractures in first-, second- and third-degree relatives - A Swedish nationwide real-world family study
Source: PLoS One. 2026 Sep 11;21(9):e0358074. doi: 10.1371/journal.pone.0358074 (PMC13567828; doi:10.1371/journal.pone.0358074)
Supplement: Supplementary file 7 [file pone.0358074.s007.docx]

| **Supplementary Table 7. Discordance analysis (after deleting patients with M80 and M81 diagnosed simultaneously or M80 at first visit followed by M81), full models. Familial Hazard ratios (HRs) with 95% confidence intervals (95% CI) for patients with osteoporosis (OP) or fractures at common osteoporotic fracture sites (=Fractures at OP sites).** | | | | |
| --- | --- | --- | --- | --- |
|  |  | Cases, No. /Persons at risk, No. | HR^$^ | 95% CI |
| **Twins**  (n=148768) | M80→M80 | 10/271 | **3.77***** | (1.51-9.40) |
|  | M81→M81 | 90/1064 | **2.74***** | (2.01-3.73) |
|  | M80 →M81 | 23/1064 | **2.55***** | (1.63-3.97) |
|  | M81 →M80 | 23/271 | **2.37***** | (1.58-3.56) |
|  | M80 → Fractures at OP sites | 65/9189 | **2.03***** | (1.52-2.71) |
|  | M81 → Fractures at OP sites | 178/9189 | **1.36***** | (1.15-1.61) |
|  |  |  |  |  |
| **Full siblings** (n=9973468) | M80→M80 | 386/22799 | **2.57***** | (2.23-2.96) |
|  | M81→M81 | 4118/ 81017 | **2.38***** | (2.26-2.51) |
|  | M80 →M81 | 900/ 81017 | **1.72***** | (1.60-1.85) |
|  | M81 →M80 | 900/22799 | **1.76***** | (1.64-1.89) |
|  | M80 → Fractures at OP sites | 3482/ 681624 | **1.38***** | (1.32-1.43) |
|  | M81 → Fractures at OP sites | 11451/ 681624 | **1.30***** | (1.27-1.32) |
|  |  |  |  |  |
| **Half siblings** (n=2837604) | M80→M80 | 20/2635 | **2.25***** | (1.47-3.43) |
|  | M81→M81 | 242/ 10459 | **1.94***** | (1.68-2.24) |
|  | M80 →M81 | 47/ 10459 | **1.38*** | (1.03-1.85) |
|  | M81 →M80 | 47/ 2635 | **1.42*** | (1.08-1.88) |
|  | M80 → Fractures at OP sites | 265/ 169180 | **1.16*** | (1.02-1.32) |
|  | M81 → Fractures at OP sites | 962/ 169180 | **1.08*** | (1.01-1.16) |
|  |  |  |  |  |
| **Cousins** (n=25846756) | M80→M80 | 6/5957 | **2.49*** | (1.11-5.58) |
|  | M81→M81 | 128/30145 | **1.99***** | (1.65-2.39) |
|  | M80 →M81 | 18/30145 | 1.46 | (0.89-2.40) |
|  | M81 →M80 | 18/5957 | 1.43 | (0.92-2.24) |
|  | M80 → Fractures at OP sites | 337/ 1206603 | **1.19**** | (1.05-1.35) |
|  | M81 → Fractures at OP sites | 1460/ 1206603 | 1.02 | (0.96-1.08) |
| ^$^Adjusted for education, birth year, obesity, chronic obstructive pulmonary disease (COPD), alcohol use disorder, rheumatoid arthritis (RA), polymyalgia rheumatica (PMR), giant cell arthritis (GCA), inflammatory bowel disease (IBD), and asthma. All calculations were based on double entry. Significance levels: * p<0.05, ** p<0.01, *** p<0.001 | | | | |
