# Supplementary material for: Familial risks of hospital treated osteoporosis and fractures in first-, second- and third-degree relatives - A Swedish nationwide real-world family study
Source: PLoS One. 2026 Sep 11;21(9):e0358074. doi: 10.1371/journal.pone.0358074 (PMC13567828; doi:10.1371/journal.pone.0358074)
Supplement: Supplementary file 8 [file pone.0358074.s008.docx]

| **Supplementary Table 8. Familial population attributable fraction (PAF) and familial Standardised incidence ratio (SIR) with 95% confidence intervals (95% CI) for osteoporosis (OP) and fractures at common osteoporotic fracture sites (=Fractures at OP sites).** | | | | |
| --- | --- | --- | --- | --- |
|  | | Cases, No./Persons at risk, No. | PAF  (95% CI) | SIR  (95% CI) |
| **Twins** |  |  |  |  |
|  | **Osteoporosis** | 178/1468 | 0.09  (0.07-0.10) | **3.65*****  (3.15-4.14) |
|  | **Fractures at OP sites** | 1252/9284 | 0.05  (0.04-0.06) | **1.61*****  (1.53-1.70) |
|  |  |  |  |  |
| **Siblings** | **Osteoporosis** | 7568 /113504 | 0.04  (0.04-0.04) | **2.44*****  (2.39-2.49) |
|  | **Fractures at OP sites** | 79308/ 688424 | 0.03  (0.03-0.03) | **1.42*****  (1.41-1.43) |
|  |  |  |  |  |
| **Half siblings** | **Osteoporosis** | 408/14096 | 0.02  (0.01-0.02) | **2.32*****  (2.10-2.54) |
|  | **Fractures at OP sites** | 12294/169898 | 0.01  (0.01-0.01) | **1.15*****  (1.13-1.17) |
|  |  |  |  |  |
| **Cousins** | **Osteoporosis** | 186/38283 | 0.003  (0.002-0.003) | **2.06*****  (1.77-2.36) |
|  | **Fractures at OP sites** | 67663/1207836 | 0.007  (0.007-0.007) | **1.14*****  (1.14-1.15) |
|  |  |  |  |  |
| All calculations were based on double-entry. Significance levels: * p<0.05, ** p<0.01, *** p<0.001 | | | | |
