# Supplementary material for: Familial risks of hospital treated osteoporosis and fractures in first-, second- and third-degree relatives - A Swedish nationwide real-world family study
Source: PLoS One. 2026 Sep 11;21(9):e0358074. doi: 10.1371/journal.pone.0358074 (PMC13567828; doi:10.1371/journal.pone.0358074)
Supplement: Supplementary file 10 [file pone.0358074.s010.docx]

| **Supplementary Table 10. Familial Hazard ratios (HRs) with 95% confidence intervals (95% CI) among twins for osteoporosis-related fractures (ICD-10 = S22, S32, S422, S525, S72) for individuals with relative history of osteoporosis fracture compared with those with non-affected relatives (n=** **149002). HRs for comorbidities are displayed.** | | | | | | |
| --- | --- | --- | --- | --- | --- | --- |
|  |  | HR(95% CI) | | | | |
| **Variables** | Ref. | **Model A** | **Model B** | **Model C** | **Model D** | **Model E** |
| **Affected** | No | **2.07*****  (1.90-2.25) | **1.81*****  (1.67-1.98) | **2.07*****  (1.90-2.25) | **1.81*****  (1.66-1.97) | **1.76*****  (1.62-1.92) |
| **Year of birth** |  | **0.98*****  (0.98-0.99) | **0.99*****  (0.98-0.99) |  | **0.99*****  (0.99-0.99) | **0.99*****  (0.99-0.99) |
| **Sex** | Male | **1.11*****  (1.06-1.15) |  | **1.11*****  (1.06-1.15) | **1.11*****  (1.06-1.16) | **1.12*****  (1.07-1.16) |
| **Education** |  |  |  |  |  |  |
| 10 – 11 y | <= 9 y or missing | **0.65*****  (0.61-0.69) |  |  | **0.85*****  (0.80-0.90) | **0.89*****  (0.85-0.95) |
| > 11 y |  | **0.58*****  (0.55-0.62) |  |  | **0.87*****  (0.82-0.92) | 0.95  (0.90-1.01) |
| **Comorbidities** |  |  |  |  |  |  |
| COPD | No | **3.34*****  (3.07-3.64) |  |  |  | **1.94*****  (1.77-2.13) |
| Alcohol use disorder | No | **2.50*****  (2.31-2.70) |  |  |  | **2.21*****  (2.05-2.39) |
| Obesity | No | **1.33*****  (1.17-1.51) |  |  |  | 1.11  (0.97-1.25) |
| RA | No | **2.30*****  (1.99-2.65) |  |  |  | **1.58*****  (1.36-1.82) |
| PMR and GCA | No | **2.47*****  (2.04-2.98) |  |  |  | **1.36****  (1.12-1.66) |
| IBD | No | **1.42*****  (1.21-1.67) |  |  |  | **1.20***  (1.02-1.41) |
| Asthma | No | **1.25*****  (1.16-1.35) |  |  |  | **1.35*****  (1.25-1.46) |
| Model A: Crude. HR, Hazard ratio. Model 1 crude.  Model B adjusted for the year of birth.  Model C adjusted for sex.  Model D adjusted for sex, education and birth year.  Model E adjusted additionally for obesity, chronic obstructive pulmonary disease (COPD), alcohol use disorder, rheumatoid arthritis (RA), polymyalgia rheumatica (PMR), giant cell arthritis (GCA), inflammatory bowel disease (IBD), and asthma.  All calculations were based on double entry. Significance levels: * p<0.05, ** p<0.01, *** p<0.001 | | | | | | |
