# Supplementary material for: Familial risks of hospital treated osteoporosis and fractures in first-, second- and third-degree relatives - A Swedish nationwide real-world family study
Source: PLoS One. 2026 Sep 11;21(9):e0358074. doi: 10.1371/journal.pone.0358074 (PMC13567828; doi:10.1371/journal.pone.0358074)
Supplement: Supplementary file 11 [file pone.0358074.s011.docx]

| **Supplementary Table 11.** **Aggregated analysis with all familial relationships included in the same model. Fully adjusted Odds ratio, Hazard ratio and** **Competing-event analysis according to Fine and Gray in family members by degree of relationship (siblings, half-siblings, and cousins in one dataset for osteoporosis (ICD-10 = M80, M81), fractures at common osteoporotic fracture sites (=Fractures at OP sites) (ICD-10 =** **S22, S32, S422, S525, S72) for individuals with relative history of osteoporosis or Fractures at OP sites compared with those with non-affected relatives.** | | | |
| --- | --- | --- | --- |
| (n = 38 831 062) | ^‡^OR (95% CI) | ^&^HR(95% CI) | ^£^HR(95% CI) |
| **Osteoporosis** |  |  |  |
| Twins affected | **3.86*****  (3.05-4.88) | **3.46*****  (2.83-4.24) | **3.41*****  (2.79-4.16) |
| Full-Siblings affected | **2.39*****  (2.29-2.49) | **2.26*****  (2.17-2.34) | **2.23*****  (2.15-2.32) |
| Half-Siblings affected | **1.94*****  (1.69-2.22) | **1.86*****  (1.41-1.91) | **1.86*****  (1.63-2.11) |
| Cousins affected | **1.68*****  (1.44-1.96) | **1.64*****  (1.41-1.91) | **1.65*****  (1.42-1.92) |
| **Fractures at OP sites** |  |  |  |
| Twins affected | **2.15*****  (1.97-2.33) | **2.16*****  (2.01-2.33) | **2.13*****  (1.98-2.30) |
| Full-Siblings affected | **1.66*****  (1.64-1.68) | **1.75*****  (1.73-1.77) | **1.73*****  (1.71-1.75) |
| Half-Siblings affected | **1.24*****  (1.21-1.27) | **1.24*****  (1.21-1.27) | **1.24*****  (1.21-1.27) |
| Cousins affected | **1.15*****  (1.14-1.16) | **1.07*****  (1.06-1.07) | **1.07*****  (1.06-1.08) |
| ^‡^Logistic regression model. ^&^Cox regression model. ^£^Competing-event analysis according to Fine and Gray.  Full model adjusted for sex, education, birth year, obesity, chronic obstructive pulmonary disease (COPD), alcohol use disorder, rheumatoid arthritis (RA), polymyalgia rheumatica (PMR), giant cell arthritis (GCA), inflammatory bowel disease (IBD), and asthma.  All calculations were based on double entry. Significance levels: * p<0.05, ** p<0.01, *** p<0.001. | | | |
