# Supplementary material for: Familial risks of hospital treated osteoporosis and fractures in first-, second- and third-degree relatives - A Swedish nationwide real-world family study
Source: PLoS One. 2026 Sep 11;21(9):e0358074. doi: 10.1371/journal.pone.0358074 (PMC13567828; doi:10.1371/journal.pone.0358074)
Supplement: Supplementary file 13 [file pone.0358074.s013.docx]

| **Supplementary Table 13. Familial Hazard ratios (HRs) with 95% confidence intervals (95% CI) with inclusion of only the two oldest full-siblings (single entry) for osteoporosis (ICD-10 = M80,M81), fractures at common osteoporotic fracture sites (=Fractures at OP sites)(ICD-10 = S22, S32, S422, S525, S72) for individuals with relative history of osteoporosis or fractures at OP sites compared with those with non-affected relatives.** n = 2301446. | | | |
| --- | --- | --- | --- |
| Variable | HR(95% CI) | | |
|  | **Model 1** | **Model 2** | **Model 3** |
| **Osteoporosis** |  |  |  |
| Affected | **7.61*****  (7.24-8.00) | **2.32*****  (2.20-2.44) | **2.24*****  (2.12-2.36) |
| **Fractures at OP sites** |  |  |  |
| Affected | **1.80*****  (1.77-1.83) | **1.56*****  (1.54-1.59) | **1.53*****  (1.51-1.56) |
| HR, Hazard ratio. Model 1 crude. Model 2 adjusted for sex, education and birth year. Model 3 adjusted additionally for obesity, chronic obstructive pulmonary disease (COPD), alcohol use disorder, rheumatoid arthritis (RA), polymyalgia rheumatica (PMR), giant cell arthritis (GCA), inflammatory bowel disease (IBD), and asthma.  All calculations were based on single entry. Significance levels: * p<0.05, ** p<0.01, *** p<0.001 | | | |
