# Supplementary material for: Familial risks of hospital treated osteoporosis and fractures in first-, second- and third-degree relatives - A Swedish nationwide real-world family study
Source: PLoS One. 2026 Sep 11;21(9):e0358074. doi: 10.1371/journal.pone.0358074 (PMC13567828; doi:10.1371/journal.pone.0358074)
Supplement: Supplementary file 14 [file pone.0358074.s014.docx]

| **Supplementary Table 14. Familial Hazard ratios (HRs) with 95% confidence intervals (95% CI) among parent-offspring for osteoporosis (OP) (ICD-10= M80,M81) and fractures at common osteoporotic fracture sites (=fractures at OP sites) (ICD-10 = S22, S32, S422, S525, S72) for individuals with parent history of OP or fractures at OP sites compared with those with non-affected relatives. All families with parents who died before 1997 were excluded. n = 5133637** | | | | | | | |
| --- | --- | --- | --- | --- | --- | --- | --- |
| Variable | Person-years,  No. | Cases, No./Persons at risk, No. | Incidence rate, cases/1000 person-years | Incidence rate ratio (95%CI) | HR(95% CI) | | |
|  |  |  |  |  | **Model 1** | **Model 2** | **Model 3** |
| **Osteoporosis** |  |  |  |  |  |  |  |
| Non-affected | 89619239 | 13897/ 4913778 | 0.16  (0.15-0.16) | 1 [Reference] | 1 [Reference] | 1 [Reference] | 1 [Reference] |
| Affected | 4663209 | 3578/ 219859 | 0.77  (0.74-0.79) | **4.95*****  (4.77-5.13) | **4.60*****  (4.41-4.76) | **2.30*****  (2.21-2.39) | **2.23*****  (2.14-2.32) |
| **Fractures at OP sites** |  |  |  |  |  |  |  |
| Non-affected | 73109990 | 186693/4174264 | 2.55  (2.54-2.57) | 1 [Reference] | 1 [Reference] | 1 [Reference] | 1 [Reference] |
| Affected | 18702049 | 62856/ 959373 | 3.36  (3.33-3.39) | **1.32*****  (1.30-1.33) | **1.31*****  (1.29-1.32) | **1.28*****  (1.27-1.29) | **1.27*****  (1.26-1.28) |
| HR, Hazard ratio. Model 1 crude. Model 2 adjusted for sex, education and birth year. Model 3 adjusted additionally for obesity, chronic obstructive pulmonary disease (COPD), alcohol use disorder, rheumatoid arthritis (RA), polymyalgia rheumatica (PMR), giant cell arthritis (GCA), inflammatory bowel disease (IBD), and asthma. All calculations were based on single entry. * p<0.05, ** p<0.01, *** p<0.001. | | | | | | | |
