# Supplementary material for: Familial risks of hospital treated osteoporosis and fractures in first-, second- and third-degree relatives - A Swedish nationwide real-world family study
Source: PLoS One. 2026 Sep 11;21(9):e0358074. doi: 10.1371/journal.pone.0358074 (PMC13567828; doi:10.1371/journal.pone.0358074)
Supplement: Supplementary file 15 [file pone.0358074.s015.docx]

| **Supplementary Table 15. Familial Hazard ratios (HRs) with 95% confidence intervals (95% CI) among full-siblings for fractures at common osteoporotic fracture sites (=fractures at OP sites) (ICD-10 = S22, S32, S422, S72) (after exclusion of individuals diagnosed with first fracture of distal radius S525) for individuals with relative history of fractures at OP sites compared with those with non-affected relatives. (n=9 447 472)** | | | | | | | |
| --- | --- | --- | --- | --- | --- | --- | --- |
| **Fractures at OP sites** | Person-years,  No. | Cases, No./Persons at risk, No. | Incidence rate, cases/1000 person-years | Incidence rate ratio (95%CI) | HR(95% CI) | | |
|  |  |  |  |  | **Model 1** | **Model 2** | **Model 3** |
| **All** |  |  |  |  |  |  |  |
| Non-affected | 172230939 | 360273/ 9054243 | 2.09  (2.08-2.10) | 1 [Reference] | 1 [Reference] | 1 [Reference] | 1 [Reference] |
| Affected | 7797858 | 32956 / 393229 | 4.23  (4.18-4.27) | **2.02*****  (2.00-2.04) | **1.95*****  (1.91-1.98) | **1.39*****  (1.36-1.41) | **1.35*****  (1.32-1.37) |
| **Male** |  |  |  |  |  |  |  |
| Non-affected | 88333047 | 194945 / 4674364 | 2.21  (2.20-2.22) | 1 [Reference] | 1 [Reference] | 1 [Reference] | 1 [Reference] |
| Affected | 3972106 | 16567 / 202520 | 4.17  (4.11-4.23) | **1.89*****  (1.86-1.92) | **1.81*****  (1.77-1.85) | **1.37*****  (1.34-1.40) | **1.33*****  (1.30-1.36) |
| **Female** |  |  |  |  |  |  |  |
| Non-affected | 83897892 | 165328/ 4379879 | 1.97  (1.96-1.98) | 1 [Reference] | 1 [Reference] | 1 [Reference] | 1 [Reference] |
| Affected | 3825752 | 16389/ 190709 | 4.28  (4.22-4.35) | **2.17*****  (2.14-2.21) | **2.10*****  (2.06-2.15) | **1.40*****  (1.37-1.43) | **1.36*****  (1.34-1.39) |
| HR, Hazard ratio. Model 1 crude. Model 2 adjusted for sex, education and birth year. Model 3 adjusted additionally for obesity, chronic obstructive pulmonary disease (COPD), alcohol use disorder, rheumatoid arthritis (RA), polymyalgia rheumatica (PMR), giant cell arthritis (GCA), inflammatory bowel disease (IBD), and asthma. All calculations were based on double-entry. Significance levels. * p<0.05, ** p<0.01, *** p<0.001. | | | | | | | |
