# Supplementary material for: Familial risks of hospital treated osteoporosis and fractures in first-, second- and third-degree relatives - A Swedish nationwide real-world family study
Source: PLoS One. 2026 Sep 11;21(9):e0358074. doi: 10.1371/journal.pone.0358074 (PMC13567828; doi:10.1371/journal.pone.0358074)
Supplement: Supplementary file 16 [file pone.0358074.s016.docx]

| **Supplementary Table 16. Hazard ratios (HRs) with 95% confidence intervals (95% CIs) among full-sublings for patients with fractures at common osteoporotic fracture sites (=fractures at OP sites ie ICD-10 codes S22, S32, S72, S422, S423, S525, S825, and S826) compared with their non-affected relatives.** | | | | | | | |
| --- | --- | --- | --- | --- | --- | --- | --- |
| Variable | Person-years,  No. | Cases, No./Persons at risk, No. | Incidence rate, cases/1000 person-years | Incidence rate ratio (95%CI) | HR (95% CI) | | |
|  |  |  |  |  | **Model 1** | **Model 2** | **Model 3** |
| **Siblings** (n = 9991510) |  |  |  |  |  |  |  |
| Non-osteoporosis fracture | 171523566 | 691554/ 9200728 | 4.03  (4.02-4.04) | 1 [Reference] | 1 [Reference] | 1 [Reference] | 1 [Reference] |
| Osteoporosis fracture | 15305991 | 99228/ 790782 | 6.48  (6.44-6.52) | **1.61*****  (1.60-1.62) | **1.58*****  (1.56-1.59) | **1.40*****  (1.39-1.41) | **1.38*****  (1.36-1.39) |
| HR, Hazard ratio. Model 1 crude. Model 2 adjusted for sex, education and birth year. Model 3 adjusted additionally for obesity, chronic obstructive pulmonary disease (COPD), alcohol use disorder, rheumatoid arthritis (RA), polymyalgia rheumatica (PMR), giant cell arthritis (GCA), inflammatory bowel disease (IBD), and asthma.. All calculations were based on double entry. Significance levels: * p<0.05, ** p<0.01, *** p<0.001.  ICD-10 codes S22, S32, S72, S422, S423, S525, S825, and S826 from the National Patient Register (NPR) were used to define both the outcome and familial exposure. Estimates are derived from Fine–Gray models. | | | | | | | |
