# Supplementary material for: Familial risks of hospital treated osteoporosis and fractures in first-, second- and third-degree relatives - A Swedish nationwide real-world family study
Source: PLoS One. 2026 Sep 11;21(9):e0358074. doi: 10.1371/journal.pone.0358074 (PMC13567828; doi:10.1371/journal.pone.0358074)
Supplement: Supplementary file 17 [file pone.0358074.s017.docx]

| **Supplementary Table 17.** **Hazard ratios (HRs) with 95% confidence intervals (95% CIs) among full-sublings for patients with fractures at common osteoporotic fracture sites (=fractures at OP sites S22, S32, S72, S422, S423, S525, S825, and S826) compared with their non-affected relatives. Only patient with ICD-10 codes W00-W19 were included i.e. fall related fractures (Slipping, tripping, stumbling and falls).** | | | | | | | |
| --- | --- | --- | --- | --- | --- | --- | --- |
| Variable | Person-years,  No. | Cases, No./Persons at risk, No. | Incidence rate, cases/1000 person-years | Incidence rate ratio (95%CI) | HR (95% CI) | | |
|  |  |  |  |  | **Model 1** | **Model 2** | **Model 3** |
| **Siblings** (n = 9991510) |  |  |  |  |  |  |  |
| Non-osteoporosis fracture | 177509700 | 527298/ 9401818 | 2.97  (2.96-2.98) | 1 [Reference] | 1 [Reference] | 1 [Reference] | 1 [Reference] |
| Osteoporosis fracture | 11493425 | 62394/ 589692 | 5.43  (5.39-5.47) | **1.83*****  (1.81-1.84) | **1.78*****  (1.76-1.81) | **1.45*****  (1.43-1.47) | **1.42*****  (1.41-1.44) |
| HR, hazard ratio. Model 1: crude. Model 2: adjusted for sex, education, and birth year. Model 3: additionally adjusted for obesity, chronic obstructive pulmonary disease (COPD), alcohol use disorder, rheumatoid arthritis (RA), polymyalgia reumatica (PMR), giant cell arthritis (GCA), inflammatory bowel disease (IBD), and asthma. All calculations were based on double entry. Significance levels: *p<0.05, p<0.01, p<0.001*. Fractures at common osteoporotic sites were identified using ICD-10 codes S22, S32, S72, S422, S423, S525, S825, and S826. Only fractures classified as fall-related were included, i.e. with fall code (ICD-10 W00–W19) recorded at the same healthcare visit. Data from the National Patient Register (NPR) were used to define both the outcome and familial exposure. Subdistribution hazard ratios were estimated using Fine–Gray models. | | | | | | | |
