# Supplementary material for: Familial risks of hospital treated osteoporosis and fractures in first-, second- and third-degree relatives - A Swedish nationwide real-world family study
Source: PLoS One. 2026 Sep 11;21(9):e0358074. doi: 10.1371/journal.pone.0358074 (PMC13567828; doi:10.1371/journal.pone.0358074)
Supplement: Supplementary file 18 [file pone.0358074.s018.docx]

| **Supplementary Table 18. Distribution of treatment groups among affected full siblings using the Swedish Prescribed Drug Register with osteoporosis with fracture (M80) or osteoporosis without fracture (M81), and combined M80/M81 diagnoses.** | | | |
| --- | --- | --- | --- |
| **Treatment**  **group** | **M80**  **n = 10810** | **MB1**  **n =39061** | **Combined M80/M81**  **n =54306** |
| Both CaD and M05B | 7132 (66.0) | 24789 (63.5) | 35531 (65.4) |
| CaD only | 1739 (16.1) | 7151 (18.3) | 9373 (17.3) |
| M05B only | 383 (3.5) | 995 (2.6) | 1514 (2.8) |
| None | 1556 (14.4) | 6126 (15.7) | 7888 (14.5) |
| **Any CaD** | 8871 (82.1) | 31940 (81.8) | 44904 (82.7) |
| **Any M05B** | 7515 (69.5) | 25784 (66.0) | 37045 (68.2) |
| Abbreviations: CaD = calcium and vitamin D; M05B = anti-osteoporosis medications (ATC code M05B). CaD was defined as dispensing of A12AX or concomitant dispensing of A12AA and A11CC on the same date of dispensing. Percentages are calculated within each column. The combined M80/M81 group includes individuals with either diagnosis and does not represent the sum of the separate M80 and M81 groups. | | | |
