# Supplementary material for: Familial risks of hospital treated osteoporosis and fractures in first-, second- and third-degree relatives - A Swedish nationwide real-world family study
Source: PLoS One. 2026 Sep 11;21(9):e0358074. doi: 10.1371/journal.pone.0358074 (PMC13567828; doi:10.1371/journal.pone.0358074)
Supplement: Supplementary file 24 [file pone.0358074.s024.docx]

|  |  |  |  |
| --- | --- | --- | --- |
| **Supplementary Fig 6.** **Complementary log-log Survival Plot of fractures at common osteoporotic fracture sites (=fractures at OP sites) estimates by familial history** **among twins, full-siblings, half-siblings and cousins for individuals with relative history of fractures at OP sites compared with those with non-affected relatives.** | | | |
