# Supplementary material for: Familial risks of hospital treated osteoporosis and fractures in first-, second- and third-degree relatives - A Swedish nationwide real-world family study
Source: PLoS One. 2026 Sep 11;21(9):e0358074. doi: 10.1371/journal.pone.0358074 (PMC13567828; doi:10.1371/journal.pone.0358074)
Supplement: Supplementary file 25 [file pone.0358074.s026.docx]

**Author response:**

**Review Comments to the Author**

Reviewer #1: In this manuscript, the authors report on the familial resemblance between the close relatives - twins,
full-siblings, half-siblings, and cousins, from the Swedish Multigeneration Register linked to the National Patient Register. The familial hazard ratios (afHRs) adjusted for birth year, sex, educational level, and comorbidities, were calculated for both OP and related fractures in each category of close relatives. While the total sample of 6,548,562 individuals is impressive, the mean age of 41 years is considered too low for this age-related disease. Yet, the highest afHR in young individuals (those born from 1977 to 2018) was 22.88 for OP in siblings. This finding might be of value to identify individuals predisposed to early-onset OP risk. Also, higher heritability for OP than for osteoporotic fractures was consistently shown by GWASs for both conditions.
This is a study by a group of experts in genetic epidemiology of fracture, with an expertise in the statistical field. Since the clinical importance of genetic determination of fracture risk is incontestable, a study of its genetic predisposition should be an interesting contribution. However, there are major concerns which to me make this work far from being complete and thus rendering any contribution to the field. There are MAJOR concerns:
First and foremost, in the era of GWAS and WGS, a study of familial resemblance / heritability of the various bone traits and skeletal health characteristics belongs to the past. It remains unclear, how the knowledge produced by this report will help us discover new genetics or provide an insight into the pathogenesis of osteoporosis or its early diagnosis.
The point that the sample were “all Swedish-born people with Swedish-born parents” doesn’t still control for possible population stratification. Ethnic Swedes constitute about 75-80% of the population today, it might be larger for those born in the 1970s, but still, self-report is not sufficient for ancestry claims. Also, Heritabilities are context-dependent; they may differ by race or ethnicity, - thus findings of one study might not be generalizable to other populations.

**Author response: Thanks for these comments that we now have addressed. However, regarding population stratification, this is a problem of genetic studies such as GWAS and WGS but not for family studies without determination of the genomic variation (without DNA). Moreover, country of birth was not self-reported. Country of birth is from the Swedish population register. We now have clarified this in the following sentence: ¨Inclusion criteria were individuals born in Sweden to Swedish born parents according to the Swedish population register.¨**

**As suggested by reviewer we acknowledge the limitation of studying the Swedish population. In the limitation section we add the following statement:¨In common with all epidemiological studies, the interpretation of results is constrained by time (1997-2018) and geographical location (Sweden), resulting in some bias in time period and location.¨**

**Reviewer 1 is right asking what is the use of family studies in the era of GWAS is and WGS? We now add a section motivating our study. We add two new references and the following sentences in the introduction: ¨In the era of GWAS and WGS the use of family studies may be questioned [34,35]. However, most diseases are the result of the interactions of multiple genes and environmental factors [34,35]. Family history captures these interactions and is free to use for the patient [34,35]. Moreover, while genetic tests and precision medicine have advanced rapidly, family history remains the "first genetic test". It provides actionable clues that help prioritize individuals for genomic screening [34,35].¨**

More technical stuff: pls. define adjustment models in the Methods. in the Discussion, ll. 408-409, definition of Model 2 and Model 3 differs from that in Tables 2 & 3.

**Author response: We now have amended this in the discussion and explained in the method section the following: ¨Model 1 was crude. Model 2 was adjusted for year of birth, sex, and level of education. Model 3 was additionally adjusted for obesity, COPD, alcohol use disorder, RA, PMR and GCA, IBD and asthma.¨ Please see the manuscript for explanation of the abbreviations the first time they are mentioned.**

**Moreover, we now add the following sentence to the methods statistical section: ¨ In an additional analysis the covariates were added stepwise: model A was crude, model B was adjusted for birthyear, model C was adjusted for sex, model D was adjusted for birthyear, sex, and education, and model E was additionally adjusted for obesity, COPD, alcohol use disorder, RA, PMR and GCA, IBD, and asthma. ¨**

Finally, a minor point:
familial risk among twins is higher compared with full-siblings; the authors find it puzzling, since full-siblings and twins share household. This suggests that an effect of intrauterine (or rather sharing-of-placenta) environment is still discernable in adult age.

**Author response. We apologize for this. We now have calculated genetical resemblance for twins (0.66) that his higher than for full-siblings (0.50). Both a genetic explanation and shared uterine environment is therefor possible. We add the following sentence in the discussion: ¨However, the average genetic resemblance for twins (0.66) is higher than for full-siblings (0.50) which might suggest a genetic explanation for the higher HR among twins than full-siblings. However, we cannot exclude the contribution of shared environment. Twins unlike full-siblings share intrauterine environment.¨**

**We now add the description of calculation of genetic resemblance among twins in the methods statistical section: ¨The average genetic resemblance for twins, both monozygotic and dizygotic, was determined as 0.66 with Weinberg’s differential method. Each relative pair was assigned their average genetic resemblance (ie. 0.66 for twin pairs, 0.5 for full-sibling pairs, 0.25 for half-sibling pairs, and 0.125 for cousin pairs).¨**

Reviewer #2: Major comments
1. Very selected osteoporosis phenotype and limited generalizability
Osteoporosis cases constitute only 0.88% of the population, with a mean age around 64 years, compared with 41 years overall. This reflects a definition based solely on primary specialist diagnoses of M80–M81, with no contribution from primary care, secondary hospital codes or osteopenia/low BMD diagnoses such as M85.x. The study therefore addresses familial risk of “specialist treated or propapbly dxa verified osteoporosis,” not the broader densitometric phenotype. This should be stated clearly in the title, abstract, methods and conclusions. If possible, the authors should perform sensitivity analyses including (a) osteopenia/other bone density disorders and (b) any position M80–M81 codes. If this cannot be done, the resulting selection and its impact on external validity need fuller discussion, including the large age gap between osteoporosis and non osteoporosis groups.

**Author response: Thanks for these comments. We now state that only hospital treated OP cases and fractures were included in the study in the title, abstract, methods, results, discussion, and conclusions. We apologize for writing in the method section that only primary OP was included. All main and secondary diagnose codes for OP and fracturs in the NPR were included. We now have amended this in the method section: ¨All patients with main or secondary ICD-10 codes in NPR for osteoporosis diagnoses were used, i.e. osteoporosis with fracture (M80) and osteoporosis without fracture (M81).¨**

**To have a more complete selection of osteoporosis related cases we also studied osteoporosis-related fractures treated in hospital. Here we have a more complete ascertainment of cases because most fractures in Sweden are treated in hospital. Thus, we did not use only main codes but also all secondary codes in the national patient register (NPR), which now is stated in the method section: ¨All patients with main or secondary ICD-10 fractures codes in NPR were included.¨**

**We have also stated the following in the limitation section: ¨The prevalence of hospital treated fractures is, however, expected to be a fairly accurate estimate as most fractures are seen in specialised health care in Sweden (i.e. emergency clinics at hospitals) with high completeness though the true number of fractures might be overestimated in NPR [48].¨**

2. Temporality of exposure and “prediction” language
In the double entry design each individual appears twice in each relative pair, once as outcome and once as exposure, and “family history” is defined by whether the co relative ever has an osteoporosis or fracture diagnosis during follow up. This means that for some pairs the index event may occur before the co relative’s diagnosis, yet the pair still contributes as “exposed.” The resulting afHRs are appropriate as measures of familial aggregation, but they do not strictly estimate the effect of having a currently affected relative on future risk.
I recommend (i) clarifying in the methods that exposure is defined over the entire follow up period and (ii) toning down predictive wording in the abstract and conclusions (“predictor for future risk”) or, if possible adding a sensitivity analysis where family history is treated as a time dependent covariate that switches from unexposed to exposed only after the first diagnosis in the predictor relative. This would align the analyses more closely with the clinical prediction framing.

**Author response: Thanks for this important comment. We now clarify in the method section that exposure is defined over the entire follow-up period, and we therefore tone down the predictive wordings throughout the manuscript. We also tone down and delete the predictive wordings throughout the manuscript. For instance in the conclusion in the abstract we now rite: ¨Heredity is associated with risk of hospital treated OP and fractures in the Swedish population. The familial risk of hospital treated OP or fractures is related to the genetic closeness of the affected relative(s).¨**

3. Age structure and need for age specific familial risks
Osteoporosis and fracture risks are highly age dependent, and cases are markedly older than non cases. The models adjust for birth year, but age does not appear to be used as the underlying time scale. The age stratified analyses use a single dichotomy at the median birth year 1976 and yield an extremely high afHR of 22.88 for osteoporosis among “younger” full siblings, which is difficult to interpret without underlying counts and may be driven by sparse data. For clinical and epidemiological interpretation, the authors should provide familial HRs by attained age bands (e.g. 20–39, 40–49, 50–59, 60–69, 70–79, ≥80 years) and consider using age as the main time axis. This would answer the key question raised by the data: how strong is familial risk at different ages, given the large age gap between affected and unaffected groups.

**Author response: Thanks. We add an analysis of age stratified familial risks among full-siblings as suggested (Tables 4 and 5). This shows a very strong age dependence of the familial risks which is typical for complex disorders. We add the following in the results section:**

**¨*Age-specific familial HRs of hospital treated OP and common osteoporotic fracture site.***

**Age-specific hazard ratios (HRs) for hospital-treated OP and major osteoporotic fracture sites among full siblings are presented in Tables 4 and 5. A pronounced age-dependent gradient in familial risk was observed, particularly for OP. The familial HRs for OP were markedly elevated at younger ages, decreasing progressively with increasing age, ranging from 125.70 (95% CI 59.44–265.84) among individuals younger than 20 years to 1.61 (95% CI 1.44–1.80) among those aged 80 years and older.**

**A similar age-dependent pattern was evident for osteoporotic fracture sites, with the notable exception of individuals aged 20–39 years. For these fracture outcomes, the familial HRs declined with increasing age, ranging from 2.34 (95% CI 2.28–2.39) in individuals younger than 20 years to 1.20 (95% CI 1.14–1.26) in those aged 80 years and older.¨**

| **Table 4. Age-specific familial hazard ratios (HRs) for the association between familial exposure among full-siblings and osteoporosis (OP) (ICD-10 codes M80 or M81).** | | | | | | | | |
| --- | --- | --- | --- | --- | --- | --- | --- | --- |
| **Age group**  **(years)** | **N**  **individuals** | **Events**  **(n)** | **HRs (95% CI)** | | | | | |
|  |  |  | **Model 1** | | **Model 2** | | **Model 3** | |
| <20 | 1651375 | 679 | 132.40 | (64.20-273.05) | 129.21 | (61.74-270.40) | 125.70 | (59.44-265.84) |
| 20–39 | 2301374 | 2997 | 25.47 | (18.56-34.94) | 25.38 | (18.46-34.88) | 24.68 | (17.95-33.91) |
| 40–49 | 1143506 | 4670 | 18.21 | (15.09-21.97) | 17.87 | (14.82-21.55) | 17.58 | (14.57-21.21) |
| 50–59 | 1472477 | 19054 | 8.23 | (7.62-8.89) | 7.95 | (7.35-8.59) | 7.31 | (6.76-7.90) |
| 60–69 | 1662876 | 42459 | 3.78 | (3.60-3.97) | 3.74 | (3.56-3.92) | 3.32 | (3.15-3.49) |
| 70–79 | 1434508 | 37769 | 2.27 | (2.16-2.39) | 2.28 | (2.16-2.39) | 2.11 | (2.01-2.23) |
| ≥80 | 325394 | 5876 | 1.66 | (1.48-1.85) | 1.68 | (1.50-1.87) | 1.61 | (1.44-1.80) |
| HR, Hazard ratio. Model 1 crude. Model 2 adjusted for sex and education. Model 3 adjusted additionally for obesity, chronic obstructive pulmonary disease (COPD), alcohol use disorder, rheumatoid arthritis (RA), polymyalgia rheumatica (PMR), giant cell arthritis (GCA), inflammatory bowel disease (IBD), and asthma. All calculations were based on double entry. Estimates are derived from models including an interaction between familial exposure and age groups (reference age group: 20–39 years). | | | | | | | | |

| **Table 5. Age-specific familial hazard ratios (HRs) for the association between familial exposure and outcome in patients with common osteoporotic (OP) fractures (=fractures at OP sites ie. ICD-10 codes S22, S32, S422, S525, S72) compared with unaffected relatives.** | | | | | | | | |
| --- | --- | --- | --- | --- | --- | --- | --- | --- |
| **Age group**  **(years)** | **N**  **individuals** | **Events**  **(n)** | **HRs (95% CI)** | | | | | |
|  |  |  | **Model 1** | | **Model 2** | | **Model 3** | |
| <20 | 1760979 | 148319 | 2.37 | (2.31-2.43) | 2.36 | (2.30-2.42) | 2.34 | (2.28-2.39) |
| 20–39 | 2232710 | 77956 | 1.07 | (1.04-1.11) | 1.07 | (1.03-1.12) | 1.06 | (1.02-1.10) |
| 40–49 | 1167616 | 67770 | 2.10 | (2.03-2.18) | 2.04 | (1.97-2.11) | 1.99 | (1.93-2.06) |
| 50–59 | 1514305 | 129441 | 2.08 | (2.04-2.13) | 2.02 | (1.97-2.06) | 1.94 | (1.90-1.98) |
| 60–69 | 1665886 | 157893 | 1.70 | (1.67-1.73) | 1.67 | (1.64-1.70) | 1.62 | (1.59-1.64) |
| 70–79 | 1358327 | 92530 | 1.40 | (1.37-1.43) | 1.39 | (1.36-1.42) | 1.36 | (1.33-1.39) |
| ≥80 | 291687 | 14515 | 1.21 | (1.15-1.27) | 1.21 | (1.15-1.27) | 1.20 | (1.14-1.26) |
| HR, Hazard ratio. Model 1 crude. Model 2 adjusted for sex and education. Model 3 adjusted additionally for obesity, chronic obstructive pulmonary disease (COPD), alcohol use disorder, rheumatoid arthritis (RA), polymyalgia rheumatica (PMR), giant cell arthritis (GCA), inflammatory bowel disease (IBD), and asthma. All calculations were based on double entry. Estimates are derived from models including an interaction between familial exposure and age groups (reference age group: 20–39 years).  **We add also the following sentence in the method statistical section: ¨Among full-siblings an age stratified analysis (<20, 20-39, 40-49, 50-59, 60-69, 70-79, and 80 years and above) was performed.¨** | | | | | | | | |

4. Choice of fracture sites and ICD codes
The fracture outcome is limited to six ICD 10 codes (S72, S32, S22, S325, S422, S525) representing hip, vertebral, pelvic, proximal humeral and distal radius fractures. Other fragility fractures (e.g. rib, humeral shaft, ankle) are excluded, and the S codes do not distinguish low from high energy trauma. The rationale for this exact set of codes should be better justified and limitations acknowledged. If feasible, a sensitivity analysis including a broader set of likely low trauma fractures would be informative. Likewise, the restriction to primary M80–M81 diagnoses should be justified and its effect on case numbers quantified.

**Author response: We also, as suggested by reviewer, added possible humeral shaft, and ankle fractures in the full-sibling analysis (Supplementary Table 16). S22 includes not only the spine but also ribs and was already included in the main analysis, which now is amended. However, this did not change the results to any major degree. However, when only including fractures due to falls (ICD-10 codes W00-W19) the familial HR was higher (Supplementary Table 17).**

**As stated above we apologize for the mistake of writing primary OP and fractures. We used not only main but also all secondary ICD-codes for OP and fractures, which now is amended in the method section.**

**We add the following in the results section:**

***¨Familial hazard ratio for 8 different hospital treated common osteoporotic fracture sites***

**The familial risk among full-siblings was similar for S22, S32, S72, S422, S423, S525, S825, and S826 compared with inclusion of only five fracture sites (S22, S32, S422, S525, S72) with afHR of 1.38 (95%CI 1.36-1.39) in model 3 (Supplementary Table 16). Moreover, with inclusion only of those with fall-related fractures (ICD-10 codes W00-W19) the familial aHR among full-siblings was slightly higher 1.42 (1.41-1.44).¨**

**We add also the following sentence in the methods statistical section: ¨Moreover, among full-siblings an analysis was performed using 8 different fracture types (S22, S32, S72, S422, S423, S525, S825, and S826) instead of five fracture types (S22, S32, S422, S525, S72).¨ ¨ In a further analysis among full-siblings patients with fracture due to falls were included (W00-W19). ¨**

**We have also done a validation study among full siblings presented in the method section as follows:**

***¨*** ***Validation of ICD-10 codes M80 and M81***

**Diagnoses of OP based on hospital records (ICD-10 codes M80 and M81) have not been specifically validated. However, the Swedish NPR is generally considered to have high validity [38,39]. To indirectly support the diagnostic accuracy, prescription data were examined among affected full siblings using the Swedish Prescribed Drug Register [42].¨**

**And in the results section we add this:**

**¨In Supplementary Table 18 prescription data were examined among affected full siblings using the Swedish Prescribed Drug Register. Overall, 37,045 (68.2%) of 54,306 full siblings with an OP diagnosis (M80 or M81) had, at some point during the follow-up period (i.e. intention to treat), been prescribed medications affecting bone structure and mineralization (Anatomical Therapeutic Chemical [ATC] code M05B). Furthermore, 44,904 (82.7%) individuals had been prescribed either a combination of vitamin D and its analogues (ATC code A11CC) and calcium (ATC code A12A), or calcium in combination with vitamin D and/or other agents (ATC code A12AX) during follow-up.¨**

5. Confounding and interpretation of “genetic” effects
The models adjust for several comorbidities (RA, COPD, IBD, etc.) that are themselves heritable and may lie on the causal pathway between genetic predisposition and osteoporosis, so adjustment may partly remove genetically mediated effects. At the same time, key lifestyle factors (smoking, physical activity, BMI, medication use) are unavailable, so shared family environment may still explain some of the observed clustering. The discussion currently attributes the familial patterns mainly to genetics. I suggest a more cautious phrasing that acknowledges both shared genes and shared environment and explicitly notes the possibility of over adjustment for heritable comorbidities and residual confounding by lifestyle.

**Author response: We now tone down in the discussion the genetics and use more cautious phrasing that acknowledges both shared genes and shared environment and explicitly notes the possibility of over adjustment for heritable comorbidities and residual confounding by lifestyle. For instance, we add in the discussion: ¨……, though we cannot rule out the contribution of shared environment.¨**

**We also write as suggested by reviewer the following in the discussion: ¨ The models adjust for several comorbid conditions (COPD, alcohol use disorder, RA, PMR and GCA, IBD, and asthma), which are themselves partially heritable and may act as intermediates on the causal pathway between genetic susceptibility and osteoporosis. Consequently, such adjustment may lead to overadjustment, potentially attenuating the estimated genetically mediated effects.¨**

6. Positioning within recent genetic literature
Several large genome wide association and polygenic risk score studies (e.g. UK Biobank) have quantified the genetic architecture of BMD and fractures, generally finding high SNP based heritability for BMD but lower for fractures. Monogenic early onset osteoporosis syndromes due to WNT1, LRP5, PLS3 and SGMS2 variants illustrate how severe, early cases can be particularly informative for gene discovery. The authors briefly cite some of this work but could integrate it more systematically, explaining how their register based familial HRs complement SNP based heritability estimates and rare variant studies, and clarifying that their strict M80–M81 definition likely captures mainly more severe or early onset cases seen in specialist care.

**Author response: We now add four references using a polygenic risk score from UK biobank. We add the following sentences in the introduction: ¨ Polygenic risk scores (PRS) for OP have previously been developed [30–33]. Using data from the UK Biobank, a large genome-wide association study (GWAS) identified 1,362 independent single-nucleotide polymorphisms (SNPs) mapping to 899 loci, of which 613 were novel. Based on these findings, a PRS was constructed that demonstrated substantial heritability for BMD and OP, whereas its predictive capacity was comparatively lower for fracture outcomes [30].**

**In the discussion we also add the following sentences: ¨The high risk of hospital treated OP among young individuals might be related to rare variants [29] or enrichment of polygenic variants and a high PRS [30,31,32,33].¨**

Minor comments
1. Introduction: I suggest rephrasing “Nowadays, there exists effective treatment for OP, both with antiresorptive drugs that inhibit osteoclasts from absorbing bone and anabolic drugs that stimulate osteoblasts to build more bone” to “There are now effective treatments for OP, including antiresorptive drugs that inhibit osteoclast mediated bone resorption and anabolic agents that stimulate osteoblast mediated bone formation.”

**Author response: Thanks. We have changed the sentence as suggest by reviewer to: ¨There are now effective treatments for OP, including antiresorptive drugs that inhibit osteoclast mediated bone resorption and anabolic agents that stimulate osteoblast mediated bone formation.¨**

2. FRAX description: FRAX can be calculated using clinical risk factors alone; femoral neck BMD is an optional input rather than an inherent “limitation.” I recommend clarifying that FRAX with BMD requires DXA access, which may limit use in some settings, instead of stating that the inclusion of BMD is itself a drawback.

**Author response: We amend, as suggested by reviewer and now write: ¨However, FRAX with BMD requires DXA access, which may limit use in some settings.¨**

3. Discussion: The sentence “Still, it might be an advantage in genetics to study the most severe cases ” is vague. It would be clearer to state that severe or early onset cases are often enriched for high penetrance variants and therefore particularly informative for genetic studies, while noting that this also means the present results mainly reflect genetics of more severe specialist treated disease.

**Author response: Thanks. We change the sentence as suggested by reviewer: ¨Severe or early onset cases are often enriched for high penetrance variants and are therefore particularly informative for genetic studies [54]. The present study mainly reflects genetics of more severe specialist treated cases. ¨**
