# Supplementary material for: Familial risks of hospital treated osteoporosis and fractures in first-, second- and third-degree relatives - A Swedish nationwide real-world family study
Source: PLoS One. 2026 Sep 11;21(9):e0358074. doi: 10.1371/journal.pone.0358074 (PMC13567828; doi:10.1371/journal.pone.0358074)
Supplement: Peer review history

# Author comment: Author response to Decision Letter 0

**DOI:** 10.1371/journal.pone.0358074.r001

**Transfer Alert**

This paper was transferred from another journal. As a result, its full editorial history (including decision letters, peer reviews and author responses) may not be present.

27 Apr 2026

# Peer review: Decision Letter 0

**DOI:** 10.1371/journal.pone.0358074.r002

**Authors:** Ken Iseri

28 May 2026

Dear Dr. Anker-Hansen,

Thank you for submitting your manuscript to PLOS ONE. After careful consideration, we feel that it has merit but does not fully meet PLOS ONE’s publication criteria as it currently stands. Therefore, we invite you to submit a revised version of the manuscript that addresses the points raised during the review process.

The manuscript has potential for publication in PLOS ONE, but substantial revisions are required before it can be considered further. The reviewers raised important concerns about phenotype definition, temporality of the familial exposure, age structure, fracture outcome definition, and interpretation of genetic versus shared environmental effects. These issues must be addressed to ensure that the analyses are described in sufficient detail and that the conclusions are fully supported by the data.

Please submit your revised manuscript by Jul 12 2026 11:59PM. If you will need more time than this to complete your revisions, please reply to this message or contact the journal office at plosone@plos.org. When you're ready to submit your revision, log on to https://www.editorialmanager.com/pone/ and select the 'Submissions Needing Revision' folder to locate your manuscript file.

- A letter that responds to each point raised by the academic editor and reviewer(s). You should upload this letter as a separate file labeled 'Response to Reviewers'.
- A marked-up copy of your manuscript that highlights changes made to the original version. You should upload this as a separate file labeled 'Revised Manuscript with Track Changes'.
- An unmarked version of your revised paper without tracked changes. You should upload this as a separate file labeled 'Manuscript'.

If you would like to make changes to your financial disclosure, please include your updated statement in your cover letter. Guidelines for resubmitting your figure files are available below the reviewer comments at the end of this letter.

If applicable, we recommend that you deposit your laboratory protocols in protocols.io to enhance the reproducibility of your results. Protocols.io assigns your protocol its own identifier (DOI) so that it can be cited independently in the future. For instructions see: https://journals.plos.org/plosone/s/submission-guidelines#loc-laboratory-protocols. Additionally, PLOS ONE offers an option for publishing peer-reviewed Lab Protocol articles, which describe protocols hosted on protocols.io. Read more information on sharing protocols at https://plos.org/protocols?utm_medium=editorial-email&utm_source=authorletters&utm_campaign=protocols.

As the corresponding author, your ORCID iD is verified in the submission system and will appear in the published article. PLOS supports the use of ORCID, and we encourage all coauthors to register for an ORCID iD and use it as well. Please encourage your coauthors to verify their ORCID iD within the submission system before final acceptance, as unverified ORCID iDs will not appear in the published article. *Only*  the individual author can complete the verification step; PLOS staff *cannot*  verify ORCID iDs on behalf of authors.

We look forward to receiving your revised manuscript.

Kind regards,

Ken Iseri

Academic Editor

PLOS One

Journal Requirements:

When submitting your revision, we need you to address these additional requirements.

1.Please ensure that your manuscript meets PLOS ONE's style requirements, including those for file naming. The PLOS ONE style templates can be found at

https://journals.plos.org/plosone/s/file?id=wjVg/PLOSOne_formatting_sample_main_body.pdf and

https://journals.plos.org/plosone/s/file?id=ba62/PLOSOne_formatting_sample_title_authors_affiliations.pdf

2. Please note that funding information should not appear in any section or other areas of your manuscript. We will only publish funding information present in the Funding Statement section of the online submission form. Please remove any funding-related text from the manuscript.

3. Thank you for stating the following financial disclosure:

“ALF Funding Region Skåne (Governmental funding) to Professor Bengt Zöller and

Swedish Research Council Funding To Professor Bengt Zöller”

Please state what role the funders took in the study.  If the funders had no role, please state: "The funders had no role in study design, data collection and analysis, decision to publish, or preparation of the manuscript."

If this statement is not correct you must amend it as needed.

Please include this amended Role of Funder statement in your cover letter; we will change the online submission form on your behalf.

4. Thank you for stating the following financial disclosure:

“ALF Funding Region Skåne (Governmental funding) to Professor Bengt Zöller and

Swedish Research Council Funding To Professor Bengt Zöller”

We note that one or more of the authors is affiliated with the funding organization, indicating the funder may have had some role in the design, data collection, analysis or preparation of your manuscript for publication; in other words, the funder played an indirect role through the participation of the co-authors. If the funding organization did not play a role in the study design, data collection and analysis, decision to publish, or preparation of the manuscript and only provided financial support in the form of authors' salaries and/or research materials, please do the following:

1. Review your statements relating to the author contributions, and ensure you have specifically and accurately indicated the role(s) that these authors had in your study. These amendments should be made in the online form.

2. Confirm in your cover letter that you agree with the following statement, and we will change the online submission form on your behalf:

“The funder provided support in the form of salaries for authors [insert relevant initials], but did not have any additional role in the study design, data collection and analysis, decision to publish, or preparation of the manuscript. The specific roles of these authors are articulated in the ‘author contributions’ section.

5. Your ethics statement should only appear in the Methods section of your manuscript. If your ethics statement is written in any section besides the Methods, please move it to the Methods section and delete it from any other section. Please ensure that your ethics statement is included in your manuscript, as the ethics statement entered into the online submission form will not be published alongside your manuscript.

6. Please include captions for your Supporting Information files at the end of your manuscript, and update any in-text citations to match accordingly. Please see our Supporting Information guidelines for more information: http://journals.plos.org/plosone/s/supporting-information.

7. If the reviewer comments include a recommendation to cite specific previously published works, please review and evaluate these publications to determine whether they are relevant and should be cited. There is no requirement to cite these works unless the editor has indicated otherwise.

Additional Editor Comments:

The reviewers raised important concerns about phenotype definition, temporality of the familial exposure, age structure, fracture outcome definition, and interpretation of genetic versus shared environmental effects.

These issues must be addressed to ensure that the analyses are described in sufficient detail and that the conclusions are fully supported by the data.

[Note: HTML markup is below. Please do not edit.]

Reviewers' comments:

Reviewer's Responses to Questions

**Comments to the Author**

1. Is the manuscript technically sound, and do the data support the conclusions?

Reviewer #1: Partly

Reviewer #2: Yes

**********

2. Has the statistical analysis been performed appropriately and rigorously?

Reviewer #1: Yes

Reviewer #2: Yes

**********

3. Have the authors made all data underlying the findings in their manuscript fully available?

The PLOS Data policy

Reviewer #1: Yes

Reviewer #2: Yes

**********

4. Is the manuscript presented in an intelligible fashion and written in standard English?

Reviewer #1: Yes

Reviewer #2: Yes

**********

Reviewer #1: In this manuscript, the authors report on the familial resemblance between the close relatives - twins,

full-siblings, half-siblings, and cousins, from the Swedish Multigeneration Register linked to the National Patient Register. The familial hazard ratios (afHRs) adjusted for birth year, sex, educational level, and comorbidities, were calculated for both OP and related fractures in each category of close relatives. While the total sample of 6,548,562 individuals is impressive, the mean age of 41 years is considered too low for this age-related disease. Yet, the highest afHR in young individuals (those born from 1977 to 2018) was 22.88 for OP in siblings. This finding might be of value to identify individuals predisposed to early-onset OP risk. Also, higher heritability for OP than for osteoporotic fractures was consistently shown by GWASs for both conditions.

This is a study by a group of experts in genetic epidemiology of fracture, with an expertise in the statistical field. Since the clinical importance of genetic determination of fracture risk is incontestable, a study of its genetic predisposition should be an interesting contribution. However, there are major concerns which to me make this work far from being complete and thus rendering any contribution to the field. There are MAJOR concerns:

First and foremost, in the era of GWAS and WGS, a study of familial resemblance / heritability of the various bone traits and skeletal health characteristics belongs to the past. It remains unclear, how the knowledge produced by this report will help us discover new genetics or provide an insight into the pathogenesis of osteoporosis or its early diagnosis.

The point that the sample were “all Swedish-born people with Swedish-born parents” doesn’t still control for possible population stratification. Ethnic Swedes constitute about 75-80% of the population today, it might be larger for those born in the 1970s, but still, self-report is not sufficient for ancestry claims. Also, Heritabilities are context-dependent; they may differ by race or ethnicity, - thus findings of one study might not be generalizable to other populations.

More technical stuff: pls. define adjustment models in the Methods. in the Discussion, ll. 408-409, definition of Model 2 and Model 3 differs from that in Tables 2 & 3.

Finally, a minor point:

familial risk among twins is higher compared with full-siblings; the authors find it puzzling, since full-siblings and twins share household. This suggests that an effect of intrauterine (or rather sharing-of-placenta) environment is still discernable in adult age.

Reviewer #2: Major comments

1. Very selected osteoporosis phenotype and limited generalizability

Osteoporosis cases constitute only 0.88% of the population, with a mean age around 64 years, compared with 41 years overall. This reflects a definition based solely on primary specialist diagnoses of M80–M81, with no contribution from primary care, secondary hospital codes or osteopenia/low BMD diagnoses such as M85.x. The study therefore addresses familial risk of “specialist treated or propapbly dxa verified osteoporosis,” not the broader densitometric phenotype. This should be stated clearly in the title, abstract, methods and conclusions. If possible, the authors should perform sensitivity analyses including (a) osteopenia/other bone density disorders and (b) any position M80–M81 codes. If this cannot be done, the resulting selection and its impact on external validity need fuller discussion, including the large age gap between osteoporosis and non osteoporosis groups.

2. Temporality of exposure and “prediction” language

In the double entry design each individual appears twice in each relative pair, once as outcome and once as exposure, and “family history” is defined by whether the co relative ever has an osteoporosis or fracture diagnosis during follow up. This means that for some pairs the index event may occur before the co relative’s diagnosis, yet the pair still contributes as “exposed.” The resulting afHRs are appropriate as measures of familial aggregation, but they do not strictly estimate the effect of having a currently affected relative on future risk.

I recommend (i) clarifying in the methods that exposure is defined over the entire follow up period and (ii) toning down predictive wording in the abstract and conclusions (“predictor for future risk”) or, if possible adding a sensitivity analysis where family history is treated as a time dependent covariate that switches from unexposed to exposed only after the first diagnosis in the predictor relative. This would align the analyses more closely with the clinical prediction framing.

3. Age structure and need for age specific familial risks

Osteoporosis and fracture risks are highly age dependent, and cases are markedly older than non cases. The models adjust for birth year, but age does not appear to be used as the underlying time scale. The age stratified analyses use a single dichotomy at the median birth year 1976 and yield an extremely high afHR of 22.88 for osteoporosis among “younger” full siblings, which is difficult to interpret without underlying counts and may be driven by sparse data. For clinical and epidemiological interpretation, the authors should provide familial HRs by attained age bands (e.g. 20–39, 40–49, 50–59, 60–69, 70–79, ≥80 years) and consider using age as the main time axis. This would answer the key question raised by the data: how strong is familial risk at different ages, given the large age gap between affected and unaffected groups.

4. Choice of fracture sites and ICD codes

The fracture outcome is limited to six ICD 10 codes (S72, S32, S22, S325, S422, S525) representing hip, vertebral, pelvic, proximal humeral and distal radius fractures. Other fragility fractures (e.g. rib, humeral shaft, ankle) are excluded, and the S codes do not distinguish low from high energy trauma. The rationale for this exact set of codes should be better justified and limitations acknowledged. If feasible, a sensitivity analysis including a broader set of likely low trauma fractures would be informative. Likewise, the restriction to primary M80–M81 diagnoses should be justified and its effect on case numbers quantified.

5. Confounding and interpretation of “genetic” effects

The models adjust for several comorbidities (RA, COPD, IBD, etc.) that are themselves heritable and may lie on the causal pathway between genetic predisposition and osteoporosis, so adjustment may partly remove genetically mediated effects. At the same time, key lifestyle factors (smoking, physical activity, BMI, medication use) are unavailable, so shared family environment may still explain some of the observed clustering. The discussion currently attributes the familial patterns mainly to genetics. I suggest a more cautious phrasing that acknowledges both shared genes and shared environment and explicitly notes the possibility of over adjustment for heritable comorbidities and residual confounding by lifestyle.

6. Positioning within recent genetic literature

Several large genome wide association and polygenic risk score studies (e.g. UK Biobank) have quantified the genetic architecture of BMD and fractures, generally finding high SNP based heritability for BMD but lower for fractures. Monogenic early onset osteoporosis syndromes due to WNT1, LRP5, PLS3 and SGMS2 variants illustrate how severe, early cases can be particularly informative for gene discovery. The authors briefly cite some of this work but could integrate it more systematically, explaining how their register based familial HRs complement SNP based heritability estimates and rare variant studies, and clarifying that their strict M80–M81 definition likely captures mainly more severe or early onset cases seen in specialist care.

Minor comments

1. Introduction: I suggest rephrasing “Nowadays, there exists effective treatment for OP, both with antiresorptive drugs that inhibit osteoclasts from absorbing bone and anabolic drugs that stimulate osteoblasts to build more bone” to “There are now effective treatments for OP, including antiresorptive drugs that inhibit osteoclast mediated bone resorption and anabolic agents that stimulate osteoblast mediated bone formation.”

2. FRAX description: FRAX can be calculated using clinical risk factors alone; femoral neck BMD is an optional input rather than an inherent “limitation.” I recommend clarifying that FRAX with BMD requires DXA access, which may limit use in some settings, instead of stating that the inclusion of BMD is itself a drawback.

3. Discussion: The sentence “Still, it might be an advantage in genetics to study the most severe cases ” is vague. It would be clearer to state that severe or early onset cases are often enriched for high penetrance variants and therefore particularly informative for genetic studies, while noting that this also means the present results mainly reflect genetics of more severe specialist treated disease.

**********

what does this mean?). If published, this will include your full peer review and any attached files.

If you choose “no”, your identity will remain anonymous but your review may still be made public.

**Do you want your identity to be public for this peer review?**  For information about this choice, including consent withdrawal, please see our Privacy Policy

Reviewer #1: No

Reviewer #2: No

**********

[NOTE: If reviewer comments were submitted as an attachment file, they will be attached to this email and accessible via the submission site. Please log into your account, locate the manuscript record, and check for the action link "View Attachments". If this link does not appear, there are no attachment files.]

To ensure your figures meet our technical requirements, please review our figure guidelines: https://journals.plos.org/plosone/s/figures

You may also use PLOS’s free figure tool, NAAS, to help you prepare publication quality figures: https://journals.plos.org/plosone/s/figures#loc-tools-for-figure-preparation.

NAAS will assess whether your figures meet our technical requirements by comparing each figure against our figure specifications.

# Author comment: Author response to Decision Letter 1

**DOI:** 10.1371/journal.pone.0358074.r003

13 Aug 2026

See Response to Reviewers submitted.

Attachment Submitted filename: Response to Reviewers PLOS1.docx

# Peer review: Decision Letter 1

**DOI:** 10.1371/journal.pone.0358074.r004

**Authors:** Ken Iseri

26 Aug 2026

Familial risks of hospital treated osteoporosis and fractures in first-, second- and third-degree relatives - A Swedish nationwide real-world family study

PONE-D-26-19576R1

Dear Dr. Anker-Hansen,

We’re pleased to inform you that your manuscript has been judged scientifically suitable for publication and will be formally accepted for publication once it meets all outstanding technical requirements.

Within one week, you’ll receive an e-mail detailing the required amendments. When these have been addressed, you’ll receive a formal acceptance letter and your manuscript will be scheduled for publication.

An invoice will be generated when your article is formally accepted. Please note, if your institution has a publishing partnership with PLOS and your article meets the relevant criteria, all or part of your publication costs will be covered. Please make sure your user information is up-to-date by logging into Editorial Manager at Editorial Manager® and clicking the ‘Update My Information' link at the top of the page. For questions related to billing, please contact billing support.

If your institution or institutions have a press office, please notify them about your upcoming paper to help maximize its impact. If they’ll be preparing press materials, please inform our press team as soon as possible -- no later than 48 hours after receiving the formal acceptance. Your manuscript will remain under strict press embargo until 2 pm Eastern Time on the date of publication. For more information, please contact onepress@plos.org.

Kind regards,

Ken Iseri

Academic Editor

PLOS One

Additional Editor Comments (optional):

Reviewers' comments:

Reviewer's Responses to Questions

**Comments to the Author**

Reviewer #1: All comments have been addressed

**********

2. Is the manuscript technically sound, and do the data support the conclusions?

Reviewer #1: Yes

**********

3. Has the statistical analysis been performed appropriately and rigorously?

Reviewer #1: Yes

**********

4. Have the authors made all data underlying the findings in their manuscript fully available?

The PLOS Data policy

Reviewer #1: No

**********

5. Is the manuscript presented in an intelligible fashion and written in standard English?

Reviewer #1: Yes

**********

Reviewer #1: no further comments from this reviewer.

The authors responded most of my comments in full, as well as the other Reviewer's points

**********

what does this mean?). If published, this will include your full peer review and any attached files.

If you choose “no”, your identity will remain anonymous but your review may still be made public.

**Do you want your identity to be public for this peer review?**  For information about this choice, including consent withdrawal, please see our Privacy Policy

Reviewer #1: No

**********

# Editor report: Acceptance letter

**DOI:** 10.1371/journal.pone.0358074.r005

**Authors:** Ken Iseri

PONE-D-26-19576R1

PLOS One

Dear Dr. Anker-Hansen,

I'm pleased to inform you that your manuscript has been deemed suitable for publication in PLOS One. Congratulations! Your manuscript is now being handed over to our production team.

At this stage, our production department will prepare your paper for publication. This includes ensuring the following:

* All references, tables, and figures are properly cited

* All relevant supporting information is included in the manuscript submission,

* There are no issues that prevent the paper from being properly typeset

You will receive further instructions from the production team, including instructions on how to review your proof when it is ready. Please keep in mind that we are working through a large volume of accepted articles, so please give us a few days to review your paper and let you know the next and final steps.

Lastly, if your institution or institutions have a press office, please let them know about your upcoming paper now to help maximize its impact. If they'll be preparing press materials, please inform our press team within the next 48 hours. Your manuscript will remain under strict press embargo until 2 pm Eastern Time on the date of publication. For more information, please contact onepress@plos.org.

You will receive an invoice from PLOS for your publication fee after your manuscript has reached the completed accept phase. If you receive an email requesting payment before acceptance or for any other service, this may be a phishing scheme. Learn how to identify phishing emails and protect your accounts at https://explore.plos.org/phishing.

If we can help with anything else, please email us at customercare@plos.org.

Thank you for submitting your work to PLOS One and supporting open access.

Kind regards,

PLOS One Editorial Office Staff

on behalf of

Dr. Ken Iseri

Academic Editor

PLOS One
